# MHCpLogics: an interactive machine learning-based tool for unsupervised data visualization and cluster analysis of immunopeptidomes

**DOI:** 10.1093/bib/bbae087

**Published:** 2024-03-14

**Authors:** Mohammad Shahbazy, Sri H Ramarathinam, Chen Li, Patricia T Illing, Pouya Faridi, Nathan P Croft, Anthony W Purcell

**Affiliations:** Department of Biochemistry and Molecular Biology and Infection and Immunity Program, Biomedicine Discovery Institute, Monash University, Melbourne, VIC 3800, Australia; Department of Biochemistry and Molecular Biology and Infection and Immunity Program, Biomedicine Discovery Institute, Monash University, Melbourne, VIC 3800, Australia; Department of Biochemistry and Molecular Biology and Infection and Immunity Program, Biomedicine Discovery Institute, Monash University, Melbourne, VIC 3800, Australia; Department of Biochemistry and Molecular Biology and Infection and Immunity Program, Biomedicine Discovery Institute, Monash University, Melbourne, VIC 3800, Australia; Centre for Cancer Research, Hudson Institute of Medical Research, Clayton, VIC 3168, Australia; Monash Proteomics and Metabolomics Platform, Department of Medicine, School of Clinical Sciences, Monash University, Clayton, VIC 3800, Australia; Department of Biochemistry and Molecular Biology and Infection and Immunity Program, Biomedicine Discovery Institute, Monash University, Melbourne, VIC 3800, Australia; Department of Biochemistry and Molecular Biology and Infection and Immunity Program, Biomedicine Discovery Institute, Monash University, Melbourne, VIC 3800, Australia

**Keywords:** Major histocompatibility complex, Human leukocyte antigens, Immunopeptidomics, Unsupervised machine learning, Data visualization, HLA peptide ligands

## Abstract

The major histocompatibility complex (MHC) encodes a range of immune response genes, including the human leukocyte antigens (HLAs) in humans. These molecules bind peptide antigens and present them on the cell surface for T cell recognition. The repertoires of peptides presented by HLA molecules are termed immunopeptidomes. The highly polymorphic nature of the genres that encode the HLA molecules confers allotype-specific differences in the sequences of bound ligands. Allotype-specific ligand preferences are often defined by peptide-binding motifs. Individuals express up to six classical class I HLA allotypes, which likely present peptides displaying different binding motifs. Such complex datasets make the deconvolution of immunopeptidomic data into allotype-specific contributions and further dissection of binding-specificities challenging. Herein, we developed MHCpLogics as an interactive machine learning-based tool for mining peptide-binding sequence motifs and visualization of immunopeptidome data across complex datasets. We showcase the functionalities of MHCpLogics by analyzing both in-house and published mono- and multi-allelic immunopeptidomics data. The visualization modalities of MHCpLogics allow users to inspect clustered sequences down to individual peptide components and to examine broader sequence patterns within multiple immunopeptidome datasets. MHCpLogics can deconvolute large immunopeptidome datasets enabling the interrogation of clusters for the segregation of allotype-specific peptide sequence motifs, identification of sub-peptidome motifs, and the exportation of clustered peptide sequence lists. The tool facilitates rapid inspection of immunopeptidomes as a resource for the immunology and vaccine communities. MHCpLogics is a standalone application available via an executable installation at: https://github.com/PurcellLab/MHCpLogics.

## Introduction

The major histocompatibility complex (MHC) encodes a set of highly polymorphic human glycoproteins termed human leukocyte antigens (HLAs). These HLA molecules bind and present peptide antigens derived from intra- and extracellular proteins at the cell surface for T cell immunosurveillance [1–4]. The antigen processing and presentation pathway that generates these peptides is central to the T cell recognition of pathogens and malignancy [5]. Different HLA allotypes can present peptides with varied sequence features and lengths. HLA class I (HLA-I) molecules bind shorter peptides (8 to 12 amino acids) derived mainly from the degradation of intracellular proteins by the proteasome. HLA class II (HLA-II) molecules can bind and present longer peptides (12 to 25 amino acids) derived from the degradation of extracellular proteins [2, 3]. The repertoires of HLA-bound peptides presented on the cell surface are termed immunopeptidomes [2–4, 6, 7].

Three genes encode the heavy chains of MHC class I molecules in humans (HLA-A, HLA-B and HLA-C). These are highly polymorphic genes, especially in the sequence encoding the peptide binding groove, with the many thousands of allotypes expressed across global populations resulting in diverse peptide binding preferences [8, 9]. The amino acids in a peptide that interact with the binding groove (pockets) of a specific HLA allotype are known as anchor residues, which are frequently at position 2 (P2) and the C-terminus (PΩ) [10, 11] of peptide ligands bound to HLA class I molecules. Thus, a given HLA allotype binds to peptides with specific sequence properties, and such sequence patterns are termed peptide-binding motifs [12, 13].

In-depth knowledge and understanding of immunopeptidomes can guide vaccine and T cell immunotherapy strategies [1, 14–16]. Mass spectrometry (MS)-based immunoproteomics can provide comprehensive datasets of peptide ligands of diverse sequence specificities isolated from multiple HLA allotypes [17]. Given that individuals can express up to six classical HLA class I allotypes [18], each with different peptide binding sequence motifs [19, 20], the deconvolution and visualization of such complex immunopeptidome data remains challenging [21, 22], highlighting a crucial demand to design and develop tools for cluster analysis of immunopeptidomes.

One of the current approaches to immunopeptidome sequence deconvolution is the *GibbsCluster* tool. This software was created as an unsupervised method for sequence alignment and cluster analysis by the Gibbs sampling strategy [23]. Peptide data with different sequence lengths are encoded and represented using a position-specific scoring matrix approach. The algorithm maximizes sequence content for individual clusters and minimizes cluster overlap [23, 24]. The *MHCcluster* tool was also introduced for cluster analysis of MHC peptides according to predicted HLA-binding specificities. *MHCcluster* generates heat maps and graphical tree-based visualizations of the bound peptides and highlights functional relationships between MHC class I and class II allotypes [25]. A number of other tools for interrogating immunopeptidomes are available: *MixMHCp* [21, 26] was developed to deconvolute MHC-I peptide data and produce peptide clusters with binding motifs. *NNAlign* [22, 27] was created for the deconvolution of poly-specific MHC peptide ligands. This tool performs unsupervised analysis of peptide data into individual clusters with unique specificities. This pipeline can also assign the clusters to an MHC allotype in an intuitive way [22, 27]. *MHCMotifDecon* [28] was designed to deconvolute immunopeptidomic datasets and designate each peptide ligand to an MHC allotype based on HLA-binding probability. This web-based supervised tool can provide sequence motifs and refine the dataset from common contaminants. Most recently, a web-based platform, termed *Immunolyser* [29], was developed to integrate *GibbsCluster* and binding prediction algorithms (i.e. *Anthem* [30], *NetMHCpan* [31, 32] and *MixMHCpred* [33]) for analysis of immunopeptidomics data, including displaying peptide length distribution, motif analysis and MHC-allotype binding prediction.

Existing tools have the capacity to perform some elements of cluster analysis and explore immunopeptidomes; however, a tool that integrates a range of complementary approaches tailored to immunopeptidomics applications is currently lacking. Here, we have developed MHCpLogics, a machine learning (ML)-based tool for peptide data analysis to explore immunopeptidomes through comprehensive approaches to visualize and deconvolute complex immunopeptidomic datasets. MHCpLogics is compatible with the output of different MS data processing software tools and search engines, allowing easy integration and peptide sequence analysis. MHCpLogics provides a method to refine HLA allotype-specific sequences and remove any unwanted endogenous or contaminant peptide signals such that they are excluded from further analysis. MHCpLogics can cluster peptide sequences across large multi-allelic datasets for data refinement, identify a specific set of peptides within the data, allow exploration of HLA-binding motifs, and export clustered peptide sequences for further analysis and reporting.

## Material and methods

### In-house experimental data from previously published studies

We used in-house datasets of HLA-I bound peptides acquired by mass spectrometry-based immunopeptidomic techniques [34]. These experimental datasets derive from cells expressing HLA-A^*^02:01 (PRIDE accession code: PXD017824 and PXD034429) [35, 36], HLA-B^*^57:01 (PXD034429) [36] and HLA-C^*^04:01 (PXD017824 and PXD034429) [37]. Notably, these datasets derive from various HLA transfectants of the C1R cell line to generate ‘mono-allelic’ HLA class I immunopeptidomes. Notably, parental C1R cells express low levels of HLA-B^*^35:03 and HLA-C^*^04:01, and peptides bound to these endogenous background allotypes are typically filtered out to generate pure mono-allelic datasets. We also utilized three published peptide datasets to assess the HLA-I peptide data visualization in this tool: (i) the micro-polymorphism datasets containing HLA-B^*^57:01 (PXD008570), -B^*^57:03 (PXD008571) and -B^*^58:01 (PXD008572) immunopeptidomes [13], (ii) two datasets of HLA-B^*^57:01 immunopeptidomes from cells cultured with or without the anti-retroviral drug abacavir (ABC) [38] and (iii) a Melanoma LM-MEL-33 cell line immunopeptidome dataset (PXD014397) [39].

### Sequence score functions

MHCpLogics provides users with two scoring functions to encode peptide sequences. The substitution matrix index (SMI) method can be utilized as a sequence score function to encode the sequence features based on analysis aims and data type. We also developed a simple sequence scoring function termed *logical-based fingerprint sequence encoding* (LFSE) to maximize the segregation of sequence clusters in transformed data space derived from machine learning algorithms for dimensionality reduction and data visualization. The LFSE algorithm logically digitizes sequences by numerical encoding to a binary vector according to specific amino acids at each position of the peptide. A list of sequences can then be treated as an input data matrix containing *‘n’* peptides. This sequence score function relies on generating a unique descriptor for each HLA-bound peptide.

### Cluster analysis algorithms

MHCpLogics integrates two agglomerative hierarchical clustering algorithms (Ward-linkage [40, 41] and centroid-linkage [42]) and *k*-means [40, 43, 44] for cluster analysis. These algorithms use only input vectors (corresponding to each observation) to make cluster inferences without referring to known or labeled outputs. We optimized algorithms and functional parameters to simplify tool usage and improve data visualization and the analysis experience.

#### Ward-linkage

MHCpLogics uses the Ward algorithm [40, 41], also called the minimum variance method, which reduces the sum of squares of errors across individual clusters. This algorithm measures the pairwise distance between individual clusters through the squared Euclidean distance of single data objects, which can be calculated as follows:


(1) \begin{equation*} d\left({x}_i,{x}_j\right)={\left\Vert{x}_i-{x}_j\right\Vert}^2 \end{equation*}

where *d*(*x_i_,x_j_*) denotes the Euclidean distance between clusters, *x_i_* and *x_j_*, representing the *i^th^* and *j^th^* clusters, respectively.

#### Centroid-linkage

We also used the centroid linkage method [41, 42, 45]). This algorithm consolidates the two closest clusters as a single cluster by means of the distance between the centroids of two individual clusters calculated as follows:


(2) \begin{equation*} d\left({x}_i,{x}_j\right)={\left\Vert{\overline{c}}_i-{\overline{c}}_j\right\Vert}^2 \end{equation*}

where *d(x_i_,x_j_)* represents the similarity distance, ${\overline{c}}_i$ and ${\overline{c}}_j$ correspond to the centroid of the *i^th^* and *j^th^* clusters, respectively, and $\left\Vert{\overline{c}}_i-{\overline{c}}_j\right\Vert$ denotes the squared Euclidean distance between the centroids of the clusters. The centroid can be defined, e.g. for *i^th^* cluster, as follows:


(3) \begin{equation*} {\overline{c}}_i=\frac{\sum_{i=1}^{Ni}{X}_i}{N_i} \end{equation*}

where *N_i_* is the number of peptides in the cluster.

#### K-means clustering

*K*-means is one of the most commonly used algorithms to group similar data objects and explore underlying sequence patterns. *K*-means defines a target number *k* of clusters and averages data points to find the number of centroids that represent each cluster. Then, it assigns every data observation to the nearest cluster while reducing the in-cluster sum of squares by keeping k as small as possible.

#### Optimization of the number of clusters

We utilized the *Calinski–Harabasz* criterion (also called the variance ratio criterion (VRC)) [46] to evaluate and optimize the number of clusters for all linkage and *k*-means algorithms. Once the algorithm output contains well-defined clusters, there is a large between-cluster variance and a small within-cluster variance where the optimal number of clusters shows the highest Calinski*–*Harabasz index value, calculated as follows:


(4) \begin{equation*} {VRC}_k=\frac{SS_B}{SS_W}\times \frac{\left(N-k\right)}{\left(k-1\right)} \end{equation*}

where *k* denotes the number of clusters, *N* represents the number of observations, *SS_B_* is the overall between-cluster variance, and *SS_W_* corresponds to the overall within-cluster variance.

### Data visualization algorithms

MHCpLogics implements unsupervised machine learning techniques for the visualization of immunopeptidomes. This visualization approach is used for the exploration and dimensionality reduction of complex peptide sequence data to help discover features of interest. In MHCpLogics, four well-established visualization methods are utilized to deconvolute large-scale immunopeptide data, including multidimensional scaling (MDS) [47], principal component analysis (PCA) [48–53], kernel PCA (kPCA) [54, 55] and *t*-distributed stochastic neighbor embedding (*t*-SNE) [56, 57].

#### Multidimensional scaling

MDS is an established approach to graphically represent relationships between peptide sequences in multidimensional data space. This algorithm transforms multivariate data into new dimensions based on the dissimilarity (e.g. Euclidean distance as default) for each pairwise comparison of objects in the original data. Then, the data objects are typically represented in two dimensions to achieve dimensionality reduction for visualization. Therefore, the distance between data objects on the newly transformed dimensions estimates their multivariate dissimilarity in the original data space.

#### Principal component analysis

PCA is a broadly-used unsupervised pattern recognition technique for dimensionality reduction and data visualization. PCA transforms high-dimensional data into fewer dimensions, keeping data information and variation as much as possible to facilitate data exploration and visualization. PCA is the basis of multivariate data analysis to project and represent data components to determine the relationships between observations and variables. The PCA algorithm aims to extract important information from the original data to express data variation as the summary coordinates called principal components (PCs) by maximizing the variance on these projected components and minimizing the residual variance. PCA utilizes eigenvalues to rank the eigenvectors to define the variance proportions of the data captured per PC. Furthermore, MHCpLogics can implement kPCA as a non-linear extension of the PCA algorithm that applies *kernel* functions.

#### T-distributed stochastic neighbor embedding

*t*-SNE is a non-linear unsupervised method widely utilized for visualization, exploration, and dimension reduction of data. The *t*-SNE algorithm measures similarity indices between every pair of objects in high-dimensional and low-dimensional data spaces to embed high-dimensional data in a two-or-three-dimensional space. It models every high-dimensional object through a two-or-three-dimensional data point so that near data points represent similar objects.

#### Programming language and software tools

MHCpLogics was written in MATLAB® version R2021a (MathWorks Inc., Natick, MA). We used AppDesigner to design the GUI of the software tool. Several released MATLAB code functions (shared over Stackoverflow, GitHub, and MATLAB File Exchange) were used to draw sequence motifs [58, 59], kPCA [60], Venn diagrams [61] and polar dendrograms [62]. We compiled and deployed the GUI by MATLAB® Compiler as a standalone executable that can be installed and run independently on Windows and Mac computers. MATLAB® Runtime version 9.10 (MathWorks Inc.) was used to run the deployed standalone application. MATLAB Runtime is required to install and run MHCpLogics, and is freely available. For benchmarking, NetMHCpan v. 4.1 [32] and GibbsCluster v. 2.0 [24] were used for HLA binding predictions and cluster analysis of immunopeptidomes, respectively. BioRender (BioRender.com) and Microsoft PowerPoint were implemented to design workflows and create schematic figures. Adobe Illustrator was also used to organize figures.

## Results

### Overview of MHCpLogics

**Figure 1:**
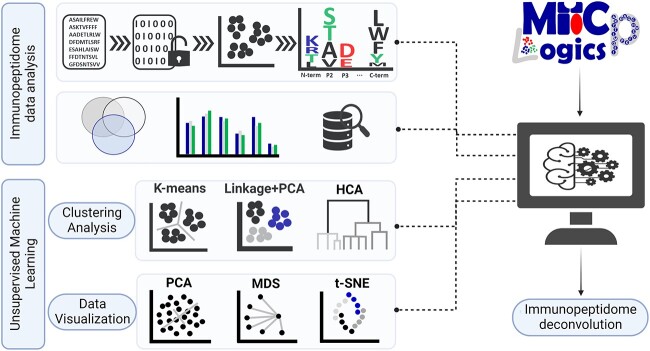
The framework of MHCpLogics. Data analysis strategies and machine learning methods used in MHCpLogics. The framework includes sequence encoding, feature selection, fundamental data analysis (e.g. Venn diagram, length distribution, amino acid prevalence and sequence motifs), proteome database search, cluster analysis and data visualization.

To analyze and visualize immunopeptidomic data, we designed a bioinformatics pipeline assisted by machine learning, termed MHCpLogics. In the MHCpLogics framework (Figure 1), we established several steps to handle and process data, including importing data, sequence scoring, peptide length-based filtering, basic analysis of peptide sequences, data preview, cluster analysis, data visualization, multi-data comparative analysis and proteome database searching to assign clustered peptides to corresponding source proteins.

**Figure 2:**
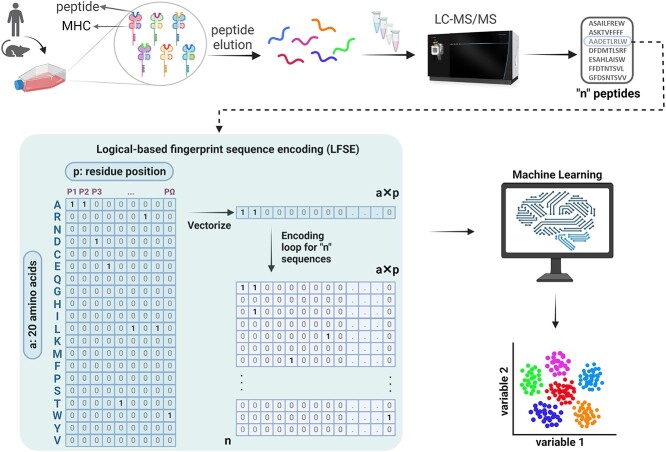
Workflow of immunopeptidome data visualization by MHCpLogics. The workflow of immunopeptidome data analysis by the MHCpLogics algorithm where the sequence scoring algorithm is detailed and termed logical-based fingerprint sequence encoding (LFSE).

To highlight the utility of MHCpLogics, we used immunopeptidomes derived from HLA-A^*^02:01, -B^*^57:01, -B^*^57:03, -B^*^58:01 and HLA-C^*^04:01 ‘mono-allelic’ datasets. Differential antigen presentation was highlighted through MHCpLogics analysis of HLA-B^*^57:01 cell line data differentially treated with the immunopeptidome modifying drug abacavir. The analysis of more complex multi-allelic data was highlighted through analysis of data derived form a patient derived a melanoma cell line LM-Mel-33. These data were utilized as case studies to assess the performance of several steps in the analytical workflow designed in MHCpLogics (Figure 2). The analytical workflow utilized to create the GUI tabs of the MHCpLogics pipeline is also detailed in Figure S1 (Supplemental Data).

MHCpLogics provides standard analyses to preview data and compare up to four datasets. Following the initial input of sequence data, it executes comparative analyses, including length distribution and amino acid frequency (AAF) at different positions of the HLA-bound peptide. It can generate Venn diagrams showing the overlap between sequences from two or three datasets. MHCpLogics also allows the definition of sequence motifs separately for each peptide length. In addition to these basic analysis methods, an ML-based strategy is then utilized to deconvolute immunopeptidome data into an optimal number of sequence-based clusters. Afterwards, users can interrogate the sequences across clusters and sub-clusters via the cluster analysis and data visualization tabs, as demonstrated in the following sections.

To deconvolute immunopeptidomes into sub-clusters (also called sub-motifs) according to the residue content at anchor and sub-anchor positions across the entire sequence, we developed the LFSE algorithm as a novel sequence scoring function. A substitution matrix index algorithm with four approaches can be used as an alternative scoring function to encode peptide sequences and analyze immunopeptidomes (Figure S2, Supplemental Data). We utilized two different datasets derived from the C1R-A^*^02:01 and C1R-B^*^57:01 expressing cell lines as an initial test analysis. We subsequently demonstrated the utility of MHCpLogics with more challenging datasets, including ‘multi-allelic’ and ‘micro-polymorphic’ HLA case studies.

### Cluster analysis and visualization of immunopeptidomes

Cluster analysis is an essential approach to deconvolute immunopeptidomes. MHCpLogics implements ML algorithms, including the linkage method and *k*-means, to cluster peptide sequence data. Combined with different sequence scoring functions, this set of algorithms enables a wide range of possibilities to examine data in-depth with two main approaches: micro-deconvolution of ‘mono-allelic’ data into sub-clusters and HLA allotype-based macro-clustering.

#### Micro-deconvolution into sub-clusters

**Figure 3:**
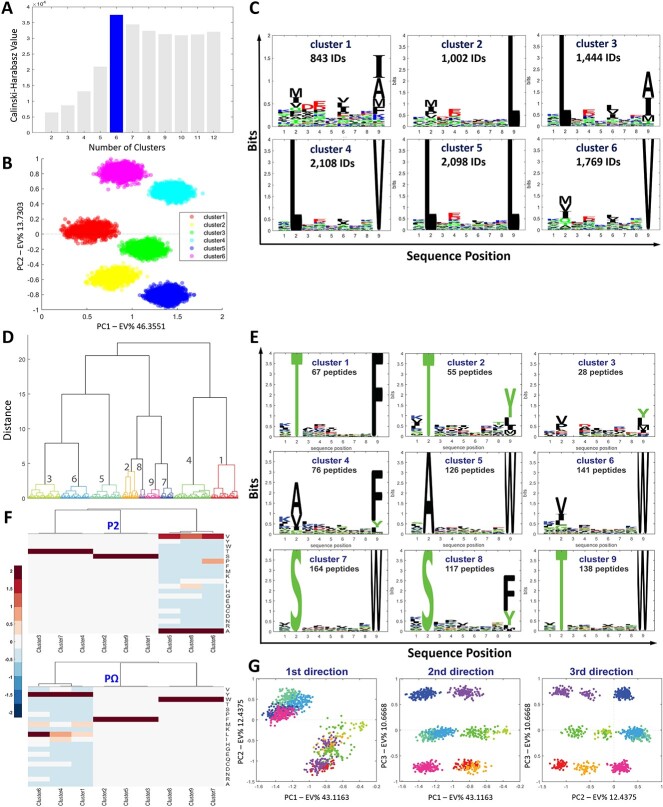
Cluster analysis of immunopeptidomes. HLA-A^*^02:01 and HLA-B^*^57:01 data were used to carry out cluster analysis and micro-deconvolution. (A) Optimization of the number of the refined C1R-A^*^02:01 peptide repertoire based on scores calculated by the *Calinski-Harabasz criterion*. (B) A scatter plot of the PCA scores on PC1 and PC2 reveals clear segregation in the peptide data space among the six clusters derived from the linkage-based clustering algorithm. (C) Sequence sub-motifs were derived from the cluster analysis of the filtered C1R-A^*^02:01 dataset, following micro-deconvolution into sub-clusters. (D) The linkage cluster analysis on the HLA-B^*^57:01 data generated a dendrogram with nine sub-clusters. (E) The corresponding sequence motifs from each of the HLA-B^*^57:01 ligand sub-clusters. (F) The dendro-heatmaps show amino acid prevalence at anchor residues across each cluster. (G) PCA plots and the corresponding peptide scores on the first three visualization directions (PC1 to PC3).

We used HLA-A^*^02:01 immunopeptidomics data generated from peptides isolated from C1R-A^*^02:01 cells to examine the cluster analysis provided by MHCpLogics and the micro-deconvolution approach in identifying sub-motifs in the dataset. After sequence encoding by LFSE and focusing specifically on 9mer HLA-I peptides (9264 IDs), the ward-linkage method (default option) was implemented to perform cluster analysis. Here, using refined HLA-A^*^02:01 binders we optimized the number of clusters, using Calinski-Harabasz criterion scores into six clusters (Figure 3A). A clear segregation among these six clusters can be observed in the peptide data space (Figure 3B). These sub-clusters showed mostly HLA-A^*^02:01 sub-motifs based on known binding preferences (Figure 3C). Subsequently, we used HLA-B^*^57:01 data (912 unique 9mer peptides), as the second case study, derived from C1R-B^*^57:01 cells after removing the C1R background. We used the same approach to optimize the number of clusters for this dataset, which resulted in nine sub-clusters. Figure 3D shows a dendrogram as an alternative way to visualize the segregation of sub-clusters. Figure 3E displays the corresponding sequence motifs for each of these sub-clusters. This analysis reveals sub-clusters of HLA-B^*^57:01 peptide ligands except for the 3^rd^ cluster, which contains 28 non-predicted HLA-B^*^57:01 binders. Another optional means of visualizing amino acid preference at anchor positions in each sub-cluster via MHCpLogics is dendro-heatmaps (Figure 3F). This strategy can integrate quantitative information with the sequence binding motifs for the analysis of positional amino acid preferences. By considering cluster analysis outcomes, MHCpLogics can also provide in-depth visualization of the peptide data space and the relationship and similarity between clusters. The first three visualization directions through peptide scores (on PC1, PC2, and PC3) (Figure 3G) reveal the color-coded clusters that correspond with those shown in the dendrogram (Figure 3D).

To assess the impact of peptide sequence scoring modalities on the performance of MHCpLogics, we compared the analysis of the mono-allelic HLA-A^*^02:01 dataset using either the SMI or LFSE scoring functions. For this case study, LFSE was a better choice to more effectively deconvolute the data into sub-motifs (Figure S3, Supplemental Data). Such, HLA-binding sub-motifs can highlight co-operative effects between different anchor residues that may be less apparent with broader, more general consensus HLA-binding motifs.

#### HLA allotype-based macro-clustering of data from multi-allelic cell lines

**Figure 4:**
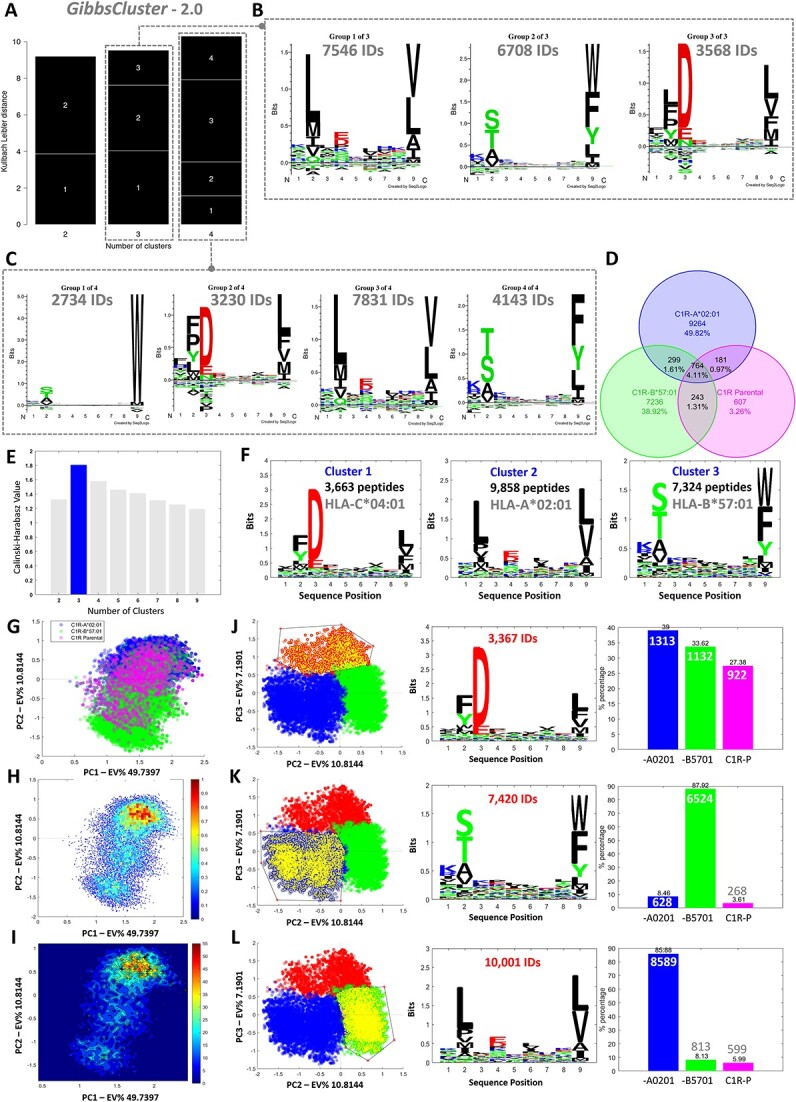
Allotype-based macro-clustering and data visualization of immunopeptidomes by MHCpLogics compared with GibbsCluster. A combined dataset of peptides eluted from C1R-A^*^02:01, C1R-B^*^57:01 and C1R Parental cells was used to compare the macro-clustering capabilities of MHCpLogics and GibbsCluster. (A) The GibbsCluster strategy revealed that the highest Kallbach Leibler distance (KLD) consisted of four clusters. (B-C) GibbsCluster was used to generate clusters of (B) three and (C) four to derive sequence motifs. (D) The same dataset was analyzed by MHCpLogics first by Venn diagram, which revealed an overlap of 764 IDs (4.11%) between these three datasets. This overlap is likely derived from peptides bound to HLA-C^*^04:01/-B^*^35:03 constitutively expressed by all three cell lines. (E) MHCpLogics showed an optimal number of three clusters as determined via the Calinski-Harabasz strategy. (F) These three clusters match the expected motifs for HLA-A^*^02:01, HLA-B^*^57:01 and HLA-C^*^04:01. (G) The scatter plot shows the peptide scores on the first direction (PC1 versus PC2) derived from the stacked data space and is color-based on individual datasets derived from different cell lines (C1R.A^*^02:01, C1R.B^*^57:01 and C1R.parental). (H-I) The density and contour plots of the HLA peptide ligands throughout the data space. (J-L) A combination of the gating strategy and ML-based data visualization of peptide data. Three gates were selected from the cluster analysis of the data to check the sequence motif and the proportion of the peptides from that subset (sub-cluster) that originated from each individual dataset.

MHCpLogics can deconvolute immunopeptidomes into allotype-specific ligandomes by macro clustering analysis. To demonstrate this, we used a stacked dataset including HLA peptidomes from unfiltered C1R-A^*^02:01, C1R-B^*^57:01 and the C1R Parental cell line, utilizing the 9mer HLA-I bound peptides from each dataset (20 845 peptides in total). The purpose of using a stacked dataset was to demonstrate the ability of the algorithm to undertake immunopeptidome data visualization and refinement, as well as perform multi-data comparative analyses to assess differences between individual peptide datasets. In this instance, we expected to observe a shared ‘background’ derived from peptides that bind to the endogenous HLA-C^*^04:01 and -B^*^35:03 allotypes expressed by parental C1R cells alongside predominant HLA-A^*^02:01- and HLA-B^*^57:01-bound peptides. As part of this analysis, we compared the performance of MHCpLogics to GibbsCluster (v. 2.0) [24], a commonly used sequence clustering tool used in the field of immunopeptidomics. Figure 4A shows that according to the GibbsCluster algorithm (combined Kallbach Leibler distance (KLD) plotted against the number of clusters applied to the dataset), the optimal number of clusters identified for this dataset was four. We then assessed the motifs for each of these clusters, as well as when restricting the number of clusters to three (expected number of main allelic-based clusters) (Figure 4B). The results showed that three clusters gave rise to the main expected motifs that corresponded to HLA-A^*^02:01 (Figure 4B, cluster 1), -B^*^57:01 (Figure 4B, cluster 2), and -C^*^04:01 (Figure 4B, cluster 3). At four clusters, the extra motif appeared to be a further deconvolution of the HLA-B^*^57:01 allotype into sequence sub-motifs (Figure 4C, clusters 1 and 4).

One of the advantages of MHCpLogics is that even though the data is analyzed as a whole, the dataset origin of each peptide is linked, enabling additional analyses. Subjecting the same dataset through MHCpLogics, we compared the datasets individually. Figure 4D shows a Venn diagram of the overlap between these three datasets. There were 764 common peptides found in all datasets (mostly HLA-HLA-C^*^04:01 ligands with a small portion of HLA-B^*^35:03 binders). Next, we compared the performance of MHCpLogics with the use of SMI and LFSE sequence scoring functions in resolving this stacked dataset into defined macro clusters. Here, the SMI approaches (especially pchcat1 and pchcat2) performed better than LFSE encoding in providing macro clusters representing specific HLA allotype ligands (Figure S4, Supplemental Data). Subsequent macro-cluster analysis was therefore carried out using the SMI approach *‘pchcat1’*. The cluster optimization, by Calinski-Harabasz strategy, showed three clear clusters of HLA peptide ligands (Figure 4D). Figure 4F shows these three clusters matched with expected motifs for HLA-A^*^02:01 (9858 IDs), HLA-B^*^57:01 (7324 IDs) and HLA-C^*^04:01 (3663 IDs) binders. In both tools, we therefore observed accurate matches between the output clusters and the known peptide binding motifs for the allotypes from which the peptide ligands were derived. However, compared to GibbsCluster, MHCpLogics provides additional visual insights into the clustered peptides, especially at the level of reporting which (and to what degree) clusters derive from each input dataset. Furthermore, an additional advantage of MHCpLogics is the speed at which this analysis was completed: MHCpLogics completed the task within 10 seconds (PC Desktop with Intel® Core™ i7–8700 CPU@3.20GHz processor and 32.0 GB Memory [RAM]), whereas GibbsCluster took approximately 15 minutes.

Next, the data visualization approach in MHCpLogics was used to explore and inspect the clusters in the peptide data space to identify and refine allotype-specific peptides; notably, GibbsCluster does not provide further analysis or exploration of the data. Figure 4G shows HLA peptide scores in the PC1-PC2 direction colored based on individual datasets, with further visualization via density and contour plots (Figure 4H-I). Although the data in Figure 4E shows the output clusters from the Calinski–Harabasz strategy, with MHCpLogics, it is possible to explore clusters manually by drawing custom gates. This is demonstrated in Figure 4J-L, where a gate was placed around each of the three main clusters (left panels), which then shows the resultant motif (middle panels) and proportion of peptides deriving from each dataset (right panels). Once again, this shows that this analysis clearly segregates the data into the anticipated HLA-C^*^04:01 (Figure 4J), -B^*^57:01 (Figure 4K) and -A^*^02:01 (Figure 4L) sequence motifs. This strategy also helps identify peptides found in more than one originating dataset (Figure 4J). Thus, MHCpLogics could deconvolve multiple stacked mono-allelic datasets to identify sequence motifs for each anticipated HLA allotype as well as highlight potential contamination or background.

### Deconvolution of HLA-bound peptide data in a multi-allelic dataset

**Figure 5:**
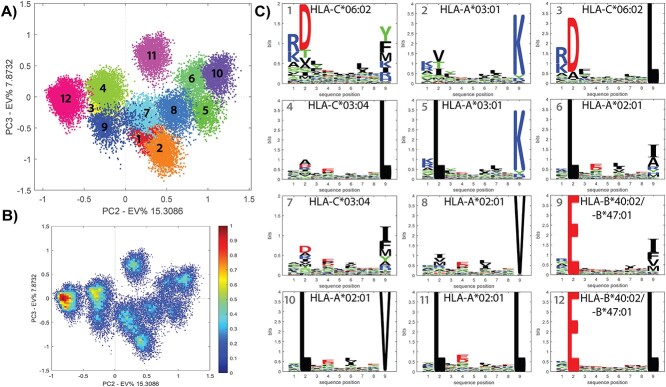
Deconvolution of multi-allelic HLA-bound peptide data. (A) Cluster analysis deconvoluted the LM-MEL-33 data into 12 sub-clusters with clear segregation. The peptide data scores on the third direction (PC2-PC3) of PCA are shown. (B) A density visualization of the peptide data scores helps to show denser clusters, containing more peptides. (C) The corresponding sequence motifs for the 12 sub-clusters. These clearly show the presence of five of the six allotypes in this cell line with two of the allotypes having indistinguishable HLA-B allotype derived peptidomes.

As shown above, MHCpLogics confers a workflow to deconvolute multi-allelic immunopeptidome data. To demonstrate this further, we used multi-allelic data derived from the patient-derived LM-MEL-33 melanoma cell line. This cell line expresses six HLA allotypes: HLA-A^*^02:01/A^*^03:01, HLA-B^*^40:02/B^*^47:01 and HLA-C^*^03:04/C^*^06:02 [39, 63]. Using the data visualization tools assisted by the cluster analysis, MHCpLogics distinguished the ligandomes of these HLA allotypes in most cases, the strong similarity of HLA-B^*^40:02 and HLA-B^*^47:01 resulted in common clusters that could not be further distinguished. Users should evaluate the different view directions (e.g. PC1/PC2, PC1/PC3 and PC2/PC3) to achieve optimal segregation of clusters. For these peptide data, this was found to be in the PC2/PC3 direction (Figure 5A), which resulted in 12 sub-clusters with excellent segregation. To examine the clusters in more detail, we used a density visualization of the peptide data scores, showing those clusters that contain a larger proportion of peptides with similar residue composition (Figure 5B).

Analysis of each sub-cluster allows for an inspection of the peptide motifs found throughout this complex dataset (Figure 5C). For instance, clusters showing sub-motifs corresponding to HLA-A^*^02:01 were identified and segregated (i.e. clusters 6, 8, 10 and 11). These HLA-A^*^02:01-derived sub-motifs in this complex multi-allelic data are similar to the four sub-motifs generated by the cluster analysis of the mono-allelic C1R-A^*^02:01 dataset shown in Figure 3G. Moreover, we can manually gate across the data space to further visualize particular sub-peptidomes (Figure S5 – Supplemental Data). Thus, MHCpLogics can deconvolute complex peptide data and provide details of the HLA-allotype-derived ligandomes in multi-allelic datasets.

### MHCpLogics-based visualization of drug induced changes in immunopeptidomes

**Figure 6:**
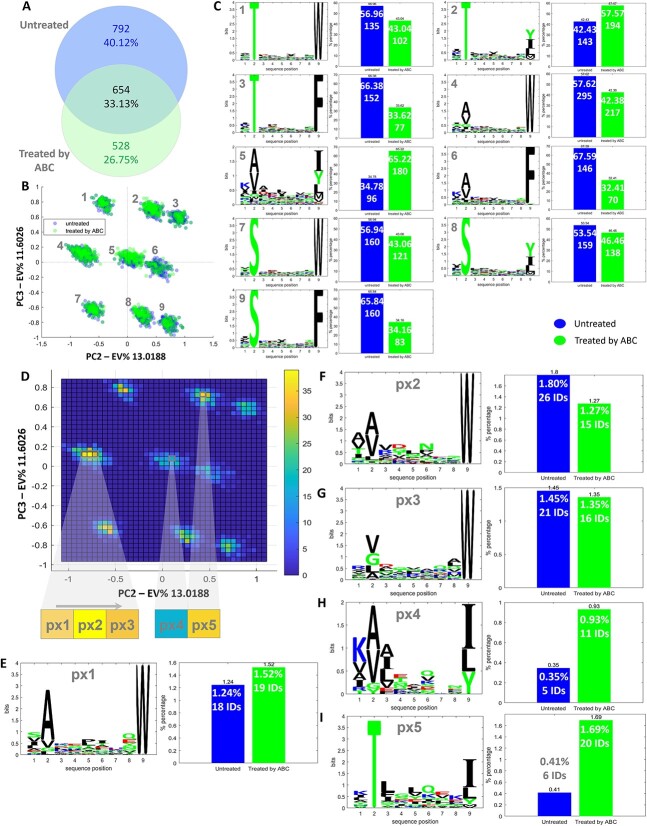
Comparative analysis of HLA-B^*^57:01 immunopeptidomes following abacavir treatment. (A) The HLA-B^*^57:01 datasets (with and without treatment by abacavir (ABC)) was analyzed by Venn diagram, which revealed the overlap between these two datasets. (B) Nine clusters (PCA analysis; PC2-PC3) are evident for the stacked dataset. (C) Sequence motifs and proportion of peptides originating from each dataset for each of the nine sub-clusters. (D) A pixel-by-pixel data visualization strategy to extract highly specific features of HLA-bound peptides is shown for five selected pixels. (E-I) Sequence motifs and proportion for peptides within each dataset (normalized internally to the total number of 9mer peptides per dataset) across the exported pixels (px1 to px5), showing differences between the immunopeptidomes derived from the culture conditions without and with ABC.

MHCpLogics enables comparative analysis across four biological/technical replicates or samples with various treatments. We used a dataset containing HLA-B^*^57:01 binders with and without treatment with abacavir, a drug that elicits an HLA-B^*^57:01 mediated hypersensitivity reaction associated with altered peptide ligand selection [38]. Figure 6A is a Venn diagram to show the overlap between these two datasets, showing considerable numbers of unique peptides for each condition and an overlap of 33.13% (654 IDs). We utilized the PCA technique assisted by the gating strategy to visualize untreated and drug-treated immunopeptidomes. In the third direction of the data space transformed by PCA (PC2-PC3), nine clusters with clear segregation exist (Figure 6B). Upon selecting each sub-cluster, we captured the corresponding sequence motif and the proportion of peptides in each sub-cluster originating from the individual datasets (Figure 6C). Abacavir can modulate the C-terminal residue preferences of HLA-B^*^57:01 bound peptides when the drug occupies the F-pocket [38], however a follow up paper that used a quantitative assessment of different classes of abacavir modulated peptide ligands demonstrated the situation is more complex than originally reported [64]. In particular, a number of kinetically distinct classes of abacavir modulated HLA-B^*^57:01 ligands were identified including peptides that were inhibited by ABC treatment, those that were unaffected, those that were facilitated (presented by HLA-B^*^57:01 of untreated cells but increased in abundance during abacavir treatment) and those that were ABC-dependent (only presented by HLA-B^*^57:01 of cells exposed to abacavir). MHCpLogics confirmed these later findings showing the spectrum of ABC-modulated peptides. For example, the fifth cluster, deconvoluted by the cluster analysis algorithm in MHCpLogics, confirmed the previous findings on the increase in the prevalence of non-canonical isoleucine (I) and leucine (L) at the C-terminus (PΩ) of peptides bound to HLA-B^*^57:01 in the presence of the drug [38]. In such analyses, isolating specific sequences at higher resolution in the data space can be helpful. MHCpLogics can visualize immunopeptides through a pixel-by-pixel view, dividing the data space into a predefined number of pixels. By clicking on each pixel, MHCpLogics the sequence motif for peptides within that specific pixel and the proportion of selected peptides originating from individual datasets (Figure 6D). In the original study [38], no abacavir-induced alteration was reported at the P2 residues of peptides restricted by HLA-B^*^57:01. Using this pixel-by-pixel data visualization strategy, we could unravel minor changes (i.e. variations of A/L/G/V residues) at P2 and even more details on the modulation at the N-terminus across three selected pixels. The fourth and fifth pixels reiterated the alterations (i.e. I and L) at PΩ residues stimulated by the ABC treatment [38]. Figures 6E-I show sequence sub-motifs and corresponding proportions (within each dataset) for these pixels of highly specific sequences isolated from the immunopeptidome data, again further highlighting MHCpLogics-guided visualization of the dataset. Notably, peptide sequences contributing to these distinctive features of the HLA-bound peptides can be exported for additional analysis or reporting.

### Visualizing the impact of micro-polymorphism on immunopeptidomes

**Figure 7:**
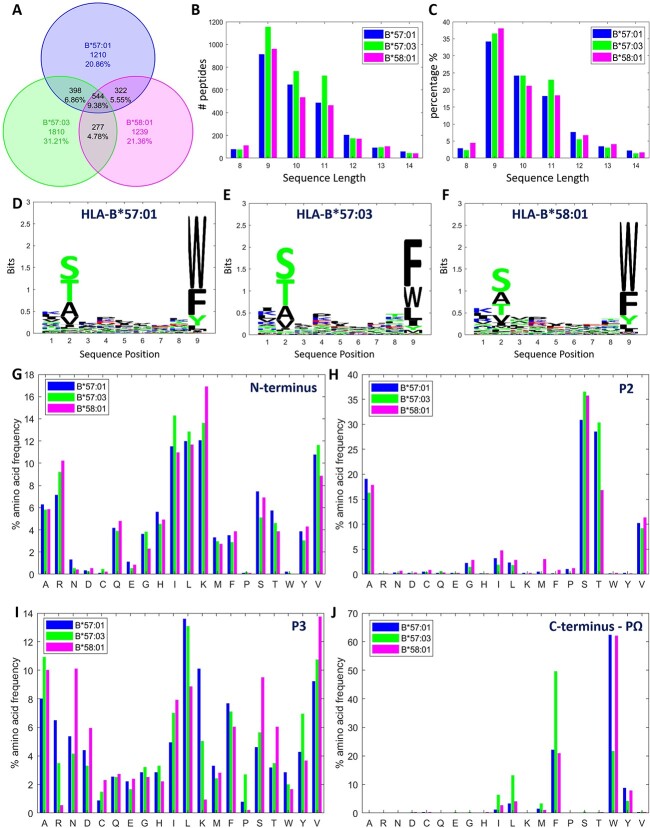
Initial comparative analysis of HLA-B57 micro-polymorphism. HLA-B57 micro-polymorphism data, containing HLA-B^*^57:01, HLA-B^*^57:03, and HLA-B^*^58:01 binders, was used to show MHCpLogics performance in basic analysis of the impact of HLA-polymorphism on immunopeptidomes. (A) A Venn diagram that demonstrates the overlap between sequences of the HLA binders (i.e. HLA-B^*^57:01, -B^*^57:03 and -B^*^58:01 peptides). (B-C) The length distribution of peptides in each dataset. (D-F) The overall sequence binding motifs for the three datasets, revealing very similar binding motifs. (G-J) The normalized amino acid frequency (AAF) at P1 (N-terminus), P2, P3 and PΩ (C-terminus)) for the peptides from each dataset.

As another challenging case study, we evaluated the capability of MHCpLogics in resolving differences in the immunopeptidomes of closely related or micro-polymorphic HLA allotypes. Micro-polymorphic HLA are defined as those allotypes that differ by just a few amino acids. Micropolymorphism can influence peptide antigen presentation, HLA stability and result in immunophenotypic diversity [13]. Since closely related HLA allotypes bind and present very similar peptide repertoires often with only subtle differences in bound peptide sequences, the comparative analysis of micro-polymorphic HLA allotypes can be demanding. We used a published dataset that explored the impact of micro-polymorphism on peptide ligands associated with three HLA-B57 supertype members. We scored peptide sequences from each dataset using the LFSE algorithm and used MHCpLogics to deconvolute and visualize the immunopeptidomes of HLA-B^*^57:01, HLA-B^*^57:03 and HLA-B^*^58:01. A Venn diagram was generated to show the overlap between sequences of the HLA binders in this dataset (Figure 7A). This analysis showed that although these datasets derive from closely related HLA allotypes, ~20–30% of peptides are unique to each dataset, with varying proportions of peptides shared between datasets (4.78 to 9.38%). We examined the peptide length distribution for each dataset (Figure 7B and C), which revealed that HLA-B^*^58:01 data possessed the highest proportion of 9mer peptides. Sequence binding motifs were generated for these three datasets individually (Figure 7D-F). These overall motifs did not demonstrate major differences in the bound peptides for each HLA allotype. MHCpLogics can assess amino acid frequency (AAF) at anchor residues and terminal positions by counting normalized proportions of amino acid residues at each position of the HLA-bound peptides. Figure 7G-J shows the AAF information at P1 (N-terminus), P2, P3 and PΩ (C-terminus) for the same data. Of note, we observed higher proportions (~60%) of tryptophan (W) at PΩ in HLA-B^*^57:01 and HLA-B^*^58:01 than in HLA-B^*^57:03 (~20%), confirming the findings in the original study [13]. Thus, we demonstrated the use of comparative analysis in MHCpLogics to assess variations at anchor residues across micro-polymorphic HLA allotypes.

**Figure 8:**
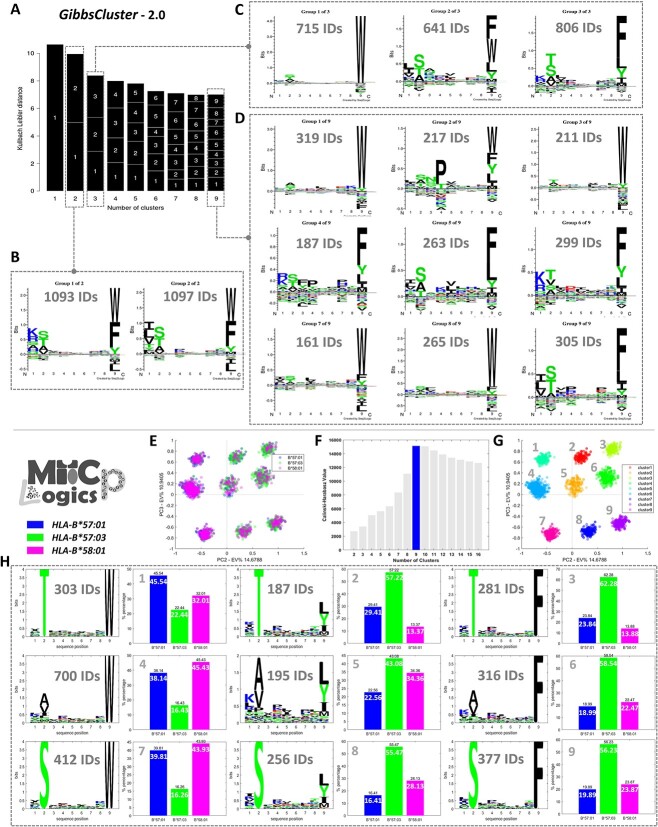
Superior cluster analysis of immunopeptidomics data from micro-polymorphic HLA-B57 allotypes by MHCpLogics. HLA-B57 micro-polymorphism data, containing 9-mer HLA-B^*^57:01, HLA-B^*^57:03, and HLA-B^*^58:01 binders, was used to compare MHCpLogics performance in dealing with subtle differences in immunopeptidomes to GibbsCluster (v. 2.0). (A) The GibbsCluster strategy showed that the highest Kallbach Leibler distance (KLD) only consisted of a single cluster. Clusters of (B) two, (C) three and (D) nine were selected to inspect the sub-motifs within each. (E) The same dataset subjected to MHCpLogics analysis revealed clear subclusters via PCA, and (F) an optimal number of nine subclusters was determined via the Calinski-Harabasz strategy. (G) A combined visualization of the clusters and the HLA-peptide scores to examine the segregation of the sub-clusters in the data space by PCA. (H) Visualization of the nine subclusters, showing the sub-motifs present, the number of peptides in each, and the proportion deriving from each dataset.

For comparison, we carried out a more in-depth analysis of the data and subjected the same data to benchmark the performance of MHCpLogics with the GibbsCluster (ver. 2.0) tool. Figure 8A shows that based on the GibbsCluster algorithm, the optimal number of clusters identified for the dataset was just one. We further assessed the motif when expanded to two, three, and nine clusters (Figure 8B-D). Although GibbsCluster is a strong tool for macro-deconvolution and allelic-based cluster analysis, its performance with micro-polymorphic HLA-B57 peptide ligands was poor, with no deconvoluted sub-motifs that provide insights into the impacts of micropolymorphism on the individual immunopeptidomes. In contrast, we assessed MHCpLogics ability to resolve the immunopeptidomes of the three HLA-B57 supertypes. Figure 8E shows the segregation of the datasets by PCA analysis (the HLA-peptide scores on PC2-PC3), with the Calinski–Harabasz strategy suggesting an optimal nine sub-clusters compared to just a single cluster observed using KLD criteria in GibbsCluster (Figure 8F). We combined the generated clusters and the HLA-peptide scores to visualize the segregation of sub-clusters (Figure 8G). Then, we used the gating strategy in MHCpLogics on each sub-cluster and conducted a comparative analysis to visualize each sub-motif and the proportion of peptides deriving from each dataset (Figure 8H). These comparative analyses highlighted differences in ligand selection between the closely related HLA-B57 allotypes. For example, the sub-motif of cluster 3 shows a prevalence of T at P2 and F at PΩ and this motif predominantly arises from HLA-B^*^57:03 binders (62.28%); in contrast, the sub-motif of cluster 7 has S at P2 and W at PΩ and this can be seen to be a predominant feature of HLA-B^*^58:01 (43.93%) and HLA-B^*^57:01 (39.81%). Collectively, these results serve to highlight the capabilities of MHCpLogics in enabling a higher-resolution analysis of closely related immunopeptidomics data derived from micro-polymorphic changes in HLA allotypes.

### Proteome database search

Mapping the feature-based selected or clustered immunopeptidome data to the proteome database is crucial in immunopeptidome analysis. MHCpLogics can execute a regular proteome database search on exported peptide sequences involved in clusters, sub-clusters and pixels. We embedded FASTA databases from UniProt to search immunopeptides derived from human and mouse origin. This database search reports protein information including: sequence, name, group, ID, peptide start and end position and accession codes. The output can be easily exported for further analysis or reporting.

### Standalone executable graphical user interface

To implement the MHCpLogics methodology and workflow, we have created a standalone executable graphical user interface (GUI) freely available at https://github.com/PurcellLab/MHCpLogics. It can be installed as a desktop tool on a local PC (^*^exe file was tested and validated on a Windows® 10 operating system). This GUI has been streamlined into step-by-step tabs: Data Import, Analysis Setting, Basic Analysis, Data Preview, Clustering Analysis, Data Visualization, Multi-Data Analysis and Proteome DB Search. The tutorial and help files are available via the GitHub repository to help users become more familiar with the use and functions of this tool. The GUI design is explained in Supplemental Data (Figures S6, S7, S8, S9, and S10) detailing different tabs and analytical workflows.

### Comparison with other immunopeptidome analysis and deconvolution tools

A few software tools have been developed for cluster analysis of HLA-I-bound peptides and are available via web servers. We compared these tools with MHCpLogics across several characteristics and capabilities in immunopeptidome data analysis. This comparison is detailed and reported in Table 1. MHCpLogics has several advantages compared with other software tools, including (i) it is an interactive pipeline, (ii) it is capable of rapid (typically within 10 s) cluster analysis even for large datasets, (iii) it provides strong data visualization methods to explore immunopeptides, (iv) it has feature selection capability and (v) it is possible to carry out multi-data comparative analysis to compare biological replicates or samples with different treatments.

**Table 1:** Benchmarking software tools for clustering analysis and deconvolution of immunopeptidomes

*Characteristics*	GibbsCluster	MHCcluster	NNAlign	MixMHCp	MHCMotifDecon	Immunolyser	MHCpLogics
*Data visualization*	No	Limited	No	No	No	Limited^a^	Yes
*Cluster analysis*	Yes	Yes	Yes	Yes	Yes	Yes (uses GibbsCluster’s algorithm)	Yes
*Interactive*	No	No	No	No	No	Yes^a^	Yes
*Multi-data comparative analysis*	No	No	No	No	No	Yes	Yes
*Macro-deconvolution for allelic-based clustering*	Yes	Yes	Yes	Yes	Yes	Yes	Yes (by using the SMI approach)
*Micro-deconvolution for analyzing micro-polymorphism*	No	NA	NA	NA	Yes	No	Yes
*Sequence Length Independent*	Yes	No	Yes	No	Yes	Yes	Yes
*(Sub)Motif-based feature selection*	No	No	No	No	No	No	Yes
*Limitation in size for uploading data*	Yes	NA	NA	NA	NA	No^b^	No^b^
*Flexible to implement different unsupervised ML algorithms by users*	NA	NA	NA	NA	NA	NA	Yes
*Assignation of a cluster to predefined allotypes (semi-supervised approach)*	Yes	Yes	Yes	No	Yes	No	No
*Web server*	Yes	Yes	Yes	Yes	Yes	Yes	In progress for the next version
*GUI standalone*	Yes (Linux)	No	No	No	No	No	Yes
*Current Tool Availability*	Yes	Yes	Yes	Yes	Yes	Yes	Yes
*Step-by-step instruction*	Yes	Yes	Yes	Yes	Yes	Yes	Yes

URL addresses for the software tools that are compared above:

GibbsCluster; https://services.healthtech.dtu.dk/service.php?GibbsCluster-2.0,

MHCcluster; https://services.healthtech.dtu.dk/service.php?MHCcluster-2.0,

NNAlign; https://services.healthtech.dtu.dk/services/NNAlign-2.1/,

MixMHCp; https://mixmhcp.vital-it.ch/#/submission,

MHCMotifDecon; https://services.healthtech.dtu.dk/services/MHCMotifDecon-1.0/,

Immunolyser; https://immunolyser.erc.monash.edu/, & MHCpLogics; https://github.com/PurcellLab/MHCpLogics.

^a^only in the case of the basic immunopeptidome analysis.

^b^not restricted but can get slow dealing with large data.

## Discussion

Modern MS-based immunopeptidomics workflows generate large-scale and complex datasets of HLA peptides, derived from cells expressing several alleles that can be challenging to analyse using existing of computational workflows and software tools. Here we introduce MHCpLogics as an interactive, fully-unsupervised data visualization and rapid cluster analysis tool to deconvolute and interrogate immunopeptidomes. MHCpLogics can deconvolute large-scale immunopeptidome data to examine clusters and sub-clusters and provide their corresponding peptide-binding sequence motifs, with easy exportation of the contributing peptide sequences. MHCpLogics is compatible with different MS data processing and computational software tools (including *Skyline* [65], *ProteinPilot™ (SCIEX)*, *Spectronaut®* [66], *MaxQuant* [67, 68], *MSFragger* [69], *DIA-NN* [70], *PEAKS™* [71] *and OpenMS/OpenSWATH* [72, 73]) and able to read their output directly for further peptide sequence analysis. MHCpLogics can handle large datasets and filter them using different approaches (e.g. specified length) without pre-processing sequences before data import. This tool performs regular immunopeptidome data analyses (e.g. Venn diagrams, length distribution, amino acid frequency at anchor residues and binding motifs) alongside data preview before cluster analysis. Users can manage and use various newly developed sequence scoring functions termed LFSE and SMI depending on research aims and data types (e.g. mono-allelic and multi-allelic clinical data).

MHCpLogics can implement different and standard ML algorithms for data visualization (i.e. PCA, *k*PCA, MDS, *t*-SNE and PCA-*t*-SNE) and cluster analysis (i.e. linkage-ward HCA, linkage-centroid HCA and *k*-means) to empower the flexible analysis of immunopeptidomes. Moreover, this tool provides data representation in a wide array of visualization options (e.g. scatter, density, contour, dendrogram and heatmap plots) and the ability to export peptide sequence lists based upon features determined during data visualization and cluster analysis. In addition, MHCpLogics allows interrogation of peptide sequence sub-motifs and we developed a new approach (Calinski*–*Harabasz strategy) to optimize the number of clusters in the immunopeptidome data. Simultaneous visualization and cluster analysis is critical for analysis and deconvolution of immunopeptidomes—the interactive nature of MHCpLogics streamlines this process. MHCpLogics is provided as an interactive software tool that has been packaged as a standalone desktop application to install and execute.

To examine and demonstrate the capabilities of MHCpLogics, we analyzed and resolved complexities in immunopeptidomes in several mono-allelic and multi-allelic datasets. Furthermore, this tool can help to refine data when there are expected contaminants (for example, endogenous shared background HLA allotypes across different cell lines). We expanded the use of MHCpLogics to demonstrate performance with more complicated multi-allelic and micro-polymorphic datasets. For the multi-allelic case study, we analyzed data from the patient-derived LM-MEL-33 melanoma cell line [39] and MHCpLogics could readily provide a comprehensive snapshot of the contribution of most HLA allotypes to the immunopeptidome. In the micro-polymorphic HLA-B57 supertype case study, MHCpLogics allowed the visualization of allotype-specific sub-motifs and provided proportional analysis of peptides originating from the individual datasets. A further feature of MHCpLogics is the ease of comparative analysis whereby small differences across biological/technical replicates or samples with various treatments can be detected using interactive data visualization techniques. Thus, distinctive HLA-I peptide ligands relevant to treatment can be assessed and identified. Moreover, MHCpLogics can execute a pixel-by-pixel data visualization strategy to export highly specific features of HLA-I peptides segregated in the data space.

The current version of MHCpLogics is not restricted by dataset size, though larger data may take longer to process depending upon the hardware the tool is operating on and the ML algorithm of choice. Even though the fully unsupervised algorithm in MHCpLogics workflow is extremely beneficial in deconvoluting data into (sub-) clusters, a limitation of the current version is that it cannot assign the clusters to predefined allotypes (this would constitute a semi-supervised approach). In future iterations of the tool, we plan to expand the algorithm to the analysis of MHC class II and nonclassical MHC I-bound peptides. Although MHCpLogics is provided as a standalone installation, we envisage that the future implementation of a web server will make this tool even more accessible to the immunopeptidomics community. In its present iteration, we anticipate that MHCpLogics will provide an essential asset to the immunological community, allowing easy and rapid visual inspection of immunopeptidomes.

**Key Points**

- MHCpLogics enables unsupervised analysis of HLA peptide ligands with the flexibility to use different machine-learning algorithms.
- An interactive and new visualization tool for immunopeptidomics data.
- Rapid cluster analysis of immunopeptidomes into allotype-specific motifs and sequence sub-motifs.
- Comparative analysis aids antigen discovery across multiple datasets.

## Supplementary Material

Supplementary_Data__revised_MHCpLogics_bbae087(1)

## Data Availability

MHCpLogics code and standalone executable application (as GUI) are freely available in the GitHub repository (https://github.com/PurcellLab/MHCpLogics). The example peptide datasets were published and deposited already in the PRIDE repository, including HLA-A^*^02:01 (PRIDE accession: PXD017824 and PXD034429) [35, 36]; HLA-B^*^57:01 (PXD034429) [36]; HLA-C^*^04:01 (PXD017824 and PXD034429) [37]; micro-polymorphism datasets containing HLA-B^*^57:01 (PXD008570), -B^*^57:03 (PXD008571), and -B^*^58:01 (PXD008572) immunopeptidomes [13]; two datasets of HLA-B^*^57:01 immunopeptidomes from cells treated with or without ABC [38]; an LM-MEL-33 dataset (PXD014397) from a Melanoma cancer cell line [39].

## Author Biographies

**Mohammad Shahbazy** is a PhD candidate in the Biomedicine Discovery Institute and Department of Biochemistry & Molecular Biology, Monash University, Australia. His research interests include bioinformatics, proteomics, immunopeptidomics, immunoinformatics, and antigen presentation.

**Sri H. Ramarathinam** is a senior scientist and mass spectrometrist addressing biomedical problems. His research interests are proteomics, cancer, and bio pharmaceutical analyses.

**Chen Li** is a research fellow at the Biomedicine Discovery Institute and the Department of Biochemistry of Molecular Biology, Monash University. His research interests include immunopeptidomics, systems proteomics, personalized medicine, and experimental bioinformatics.

**Patricia T. Illing** is a Group Leader in the Biomedicine Discovery Institute, Monash University, Australia. Her research centres on understanding peptide and small molecule presentation by MHC, and MHC-like, molecules.

**Pouya Faridi** is the head of Translational Antigen Discovery laboratory at Monash University/Hudson Institute of Medical Research, Australia. His research interests are immunopeptidomics, peptidomics, and cancer immunotherapy.

**Nathan P. Croft** is a Senior Lecturer and Group Leader at Monash University, Australia. His research focuses on understanding and quantifying the diversity of peptides displayed by major histocompatibility complex (MHC) molecules on the surface of cells.

**Anthony W. Purcell** is an NHMRC Investigator Fellow and Professor in the Department of Biochemistry & Molecular Biology, Monash University, Australia. He specialises in the identification of targets of the immune response and host-pathogen interactions.
