# Supplementary material for: MHCpLogics: an interactive machine learning-based tool for unsupervised data visualization and cluster analysis of immunopeptidomes
Source: Brief Bioinform. 2024 Mar 14;25(2):bbae087. doi: 10.1093/bib/bbae087 (PMC10940831; doi:10.1093/bib/bbae087)
Supplement: Supplementary_Data__revised_MHCpLogics_bbae087(1) [file supplementary_data__revised_mhcplogics_bbae087(1).docx]

**MHCpLogics: an interactive machine learning-based tool for unsupervised data visualization and cluster analysis of immunopeptidomes**

Mohammad Shahbazy ^1^, Sri H. Ramarathinam ^1^, Chen Li ^1^, Patricia T. Illing ^1^, Pouya Faridi ^2,3,^*, Nathan P. Croft ^1,^*, and Anthony W. Purcell ^1,^*

^1^ Department of Biochemistry and Molecular Biology and Infection and Immunity Program, Biomedicine Discovery Institute, Monash University, Melbourne, VIC 3800, Australia

^2^ Centre for Cancer Research, Hudson Institute of Medical Research, Clayton, VIC 3168, Australia

^3^ Monash Proteomics and Metabolomics Platform, Department of Medicine, School of Clinical Sciences, Monash University, Clayton, VIC 3800, Australia

* To whom correspondence should be addressed. Tel: +61 3 9902 9265; Fax: +61 3 9905 5645; Email: [anthony.purcell@monash.edu](mailto:anthony.purcell@monash.edu) (A.W.P.).

** Correspondence may also be addressed to [nathan.croft@monash.edu](mailto:nathan.croft@monash.edu) (N.P.C.); [pouya.faridi@monash.edu](mailto:pouya.faridi@monash.edu) (P.F.).

**Supplementary Data and Material**

**Software availability:**

<https://github.com/PurcellLab/MHCpLogics>

**Example Datasets:**

<https://github.com/PurcellLab/MHCpLogics/tree/main/Example%20Data>

**Instructions and Tutorial:**

<https://github.com/PurcellLab/MHCpLogics/tree/main>

| **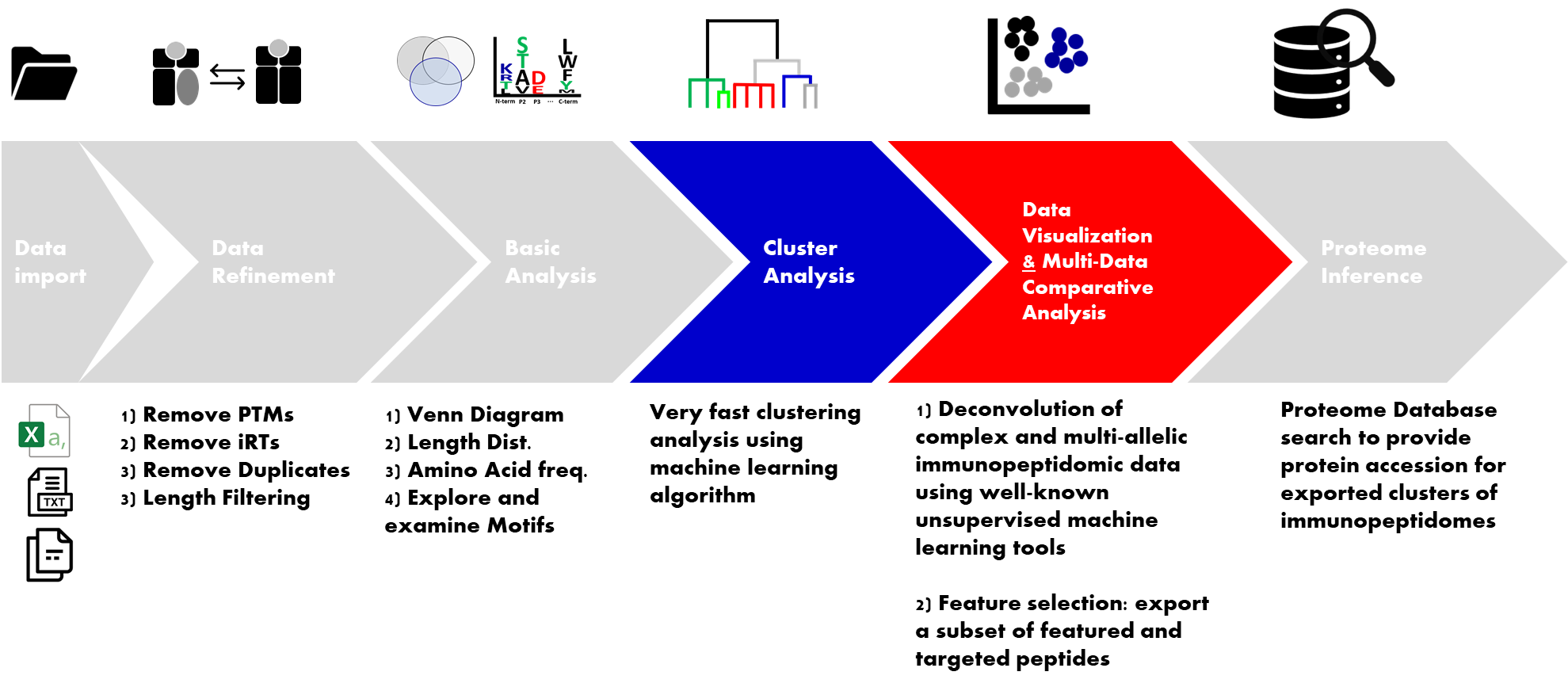** |
| --- |
| **Figure S1. Analytical workflow of MHCpLogics software tool.** The workflow used to design the GUI of the MHCpLogics pipeline with multiple tabs, including data import, data refinement, basic analysis (and data preview), cluster analysis, data visualization, multi-data comparative analysis, and proteome inference. |

**Substitution matrix index scoring function**

We used the substitution matrix index (SMI) scoring function to encode physicochemical characteristics-based residue groups. We used physicochemical features of amino acid residue-based sequences (i.e., hydrophobicity, isoelectric point, pKa at C-terminus (-COOH), pKb at N-terminus (-NH3), and molecular weight (MW)) termed as “*pchem*” approach.

Moreover, we utilized SMI-based scoring functions for encoding sequences based on physicochemical categories of residues characteristics with three categorical rules. In the first rule for the SMI method, termed “*pchcat1*”, amino acids are grouped according to the characteristics of the side chains. In the first categorical strategy, per sequence, the code substitutes the aliphatic amino acids, i.e., Alanine, Glycine, Isoleucine, Leucine, Proline, and Valine, with value of one. This function replaces aromatic (Phenylalanine, Tryptophan, and Tyrosine), acidic (Aspartic acid and Glutamic acid), basic (Arginine, Histidine, and Lysine), hydroxylic (Serine and Threonine), sulphur-containing (Cysteine and Methionine), and amidic residues (Asparagine and Glutamine), with a value of two to seven, respectively.

In the second SMI category, the code substitutes the polar amino acids, i.e., Asparagine, Cysteine, Glutamine, Glycine, Serine, Threonine, and Tyrosine, with a value of one. This function replaces hydrophobic (Alanine, Isoleucine, Leucine, Methionine, Phenylalanine, Proline, Tryptophan, and Valine), acidic (Aspartic acid, and Glutamic acid), and basic (Arginine, Histidine, and Lysine) with two to four, respectively. In the third categorical rule, we replace aromatic (Phenylalanine, Tryptophan, and Tyrosine), hydrophobic (Alanine, Isoleucine, Leucine, Methionine, Valine, and Glycine), Special (Cysteine and Proline), polar uncharged (Serine, Threonine, Asparagine, and Glutamine), and charged (Arginine, Histidine, Lysine, Aspartic acid, and Glutamic acid) with a value of one to five, respectively (Figure S3, Supplemental Data).

| 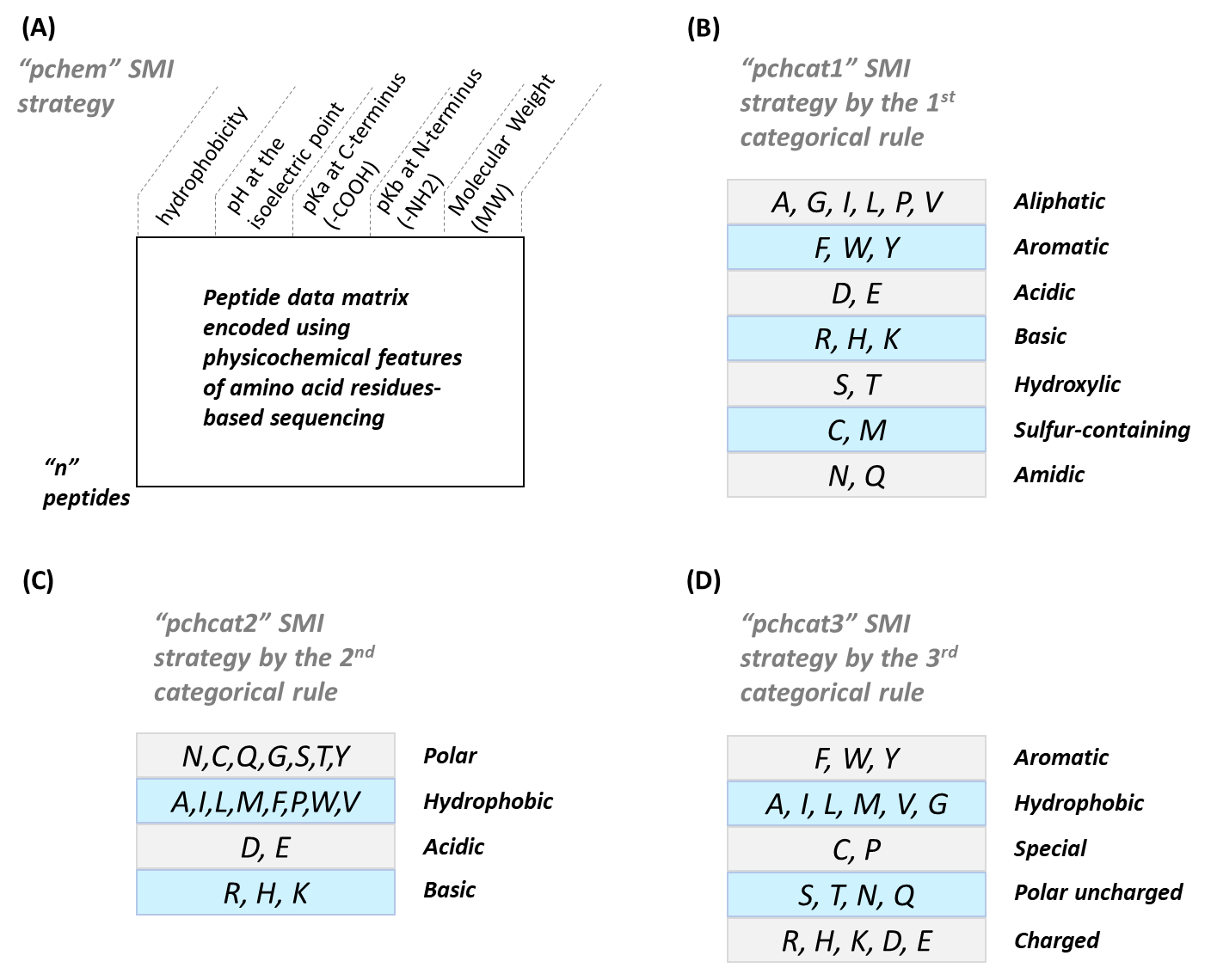 |
| --- |
| **Figure S2. Approaches for substitution matrix index (SMI) method for sequence scoring functions. (A**) Physicochemical features of amino acid residues-based sequencing (i.e., pKa, hydrophobicity, isoelectric point, pKa at C-terminus (-COOH), pKb at N-terminus (-NH2), and Molecular Weight (MW)) as termed “*pchem*”. SMI-based scoring functions are used to encode sequences based on physicochemical categories of residue characteristics with three categorical rules termed; **(B)** *phccat1*, **(C)** *pchcat2*, and **(D)** *pchcat3*. |

| 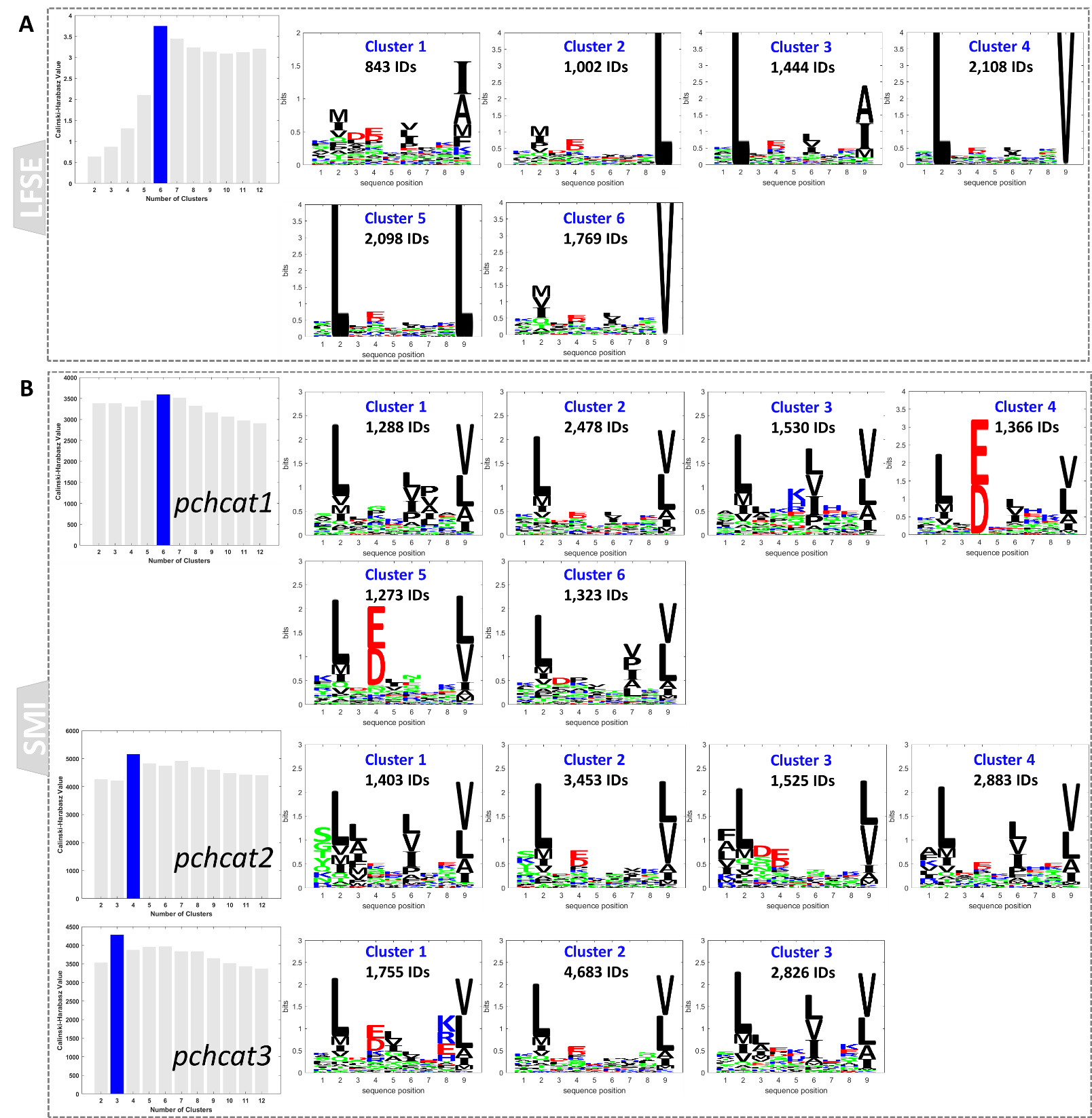 |
| --- |
| **Figure S3. Comparison of sequence scoring functions in micro clustering of mono allelic HLA-A*02:01 peptide data. (A)** The use of LFSE in MHCpLogics to perform micro cluster analysis and deconvolution into sub-clusters, resulted in six sub-motifs. **(B)** The utilization of three approaches (i.e., *pchcat1*, *pchcat2*, and *pchcat3*) of the SMI function to show MHCpLogics performance in micro deconvolution of immunopeptidomes. |

| 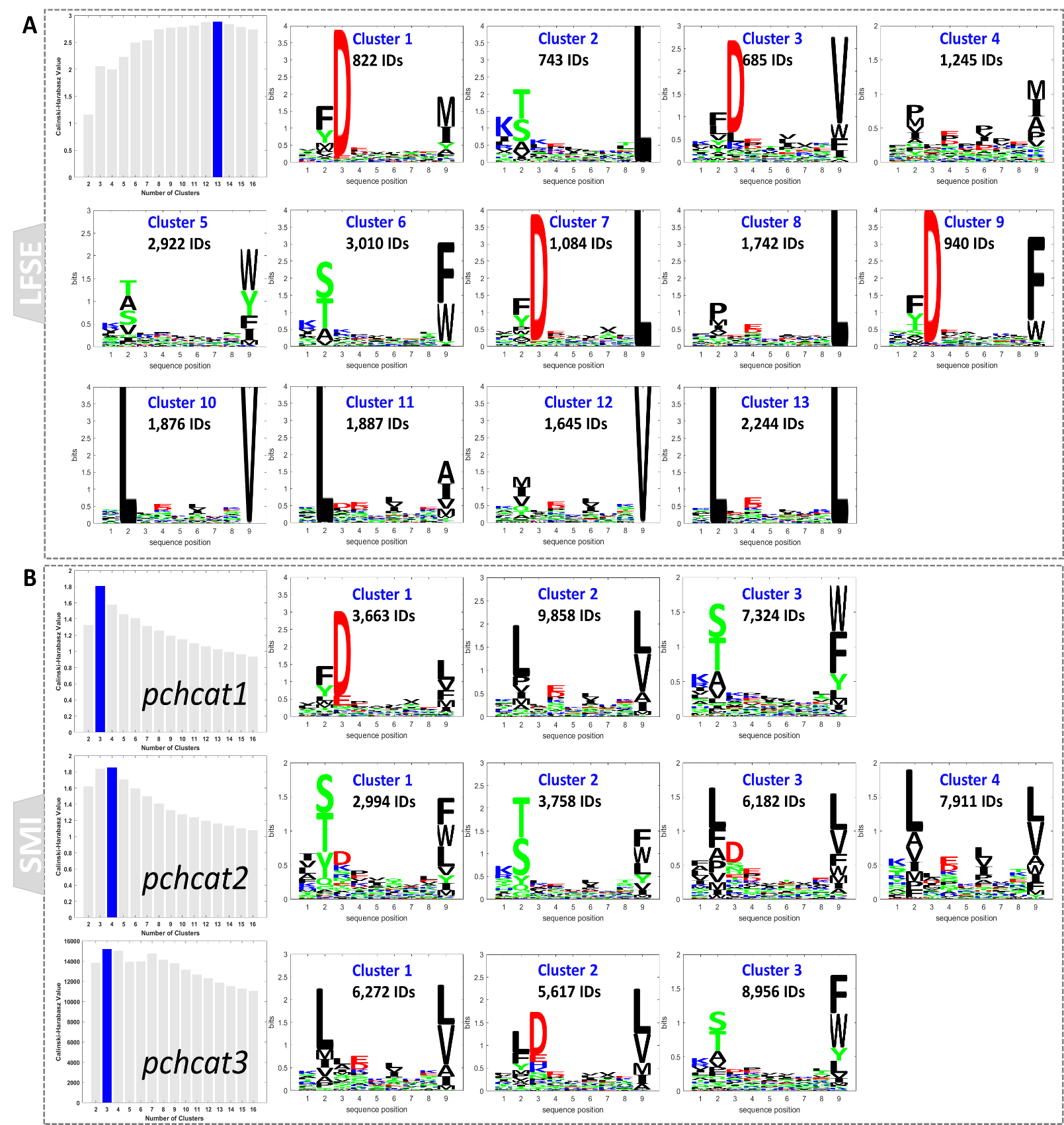 |
| --- |
| **Figure S4. Comparison of sequence scoring functions in allelic-based macro clustering of datasets containing peptides derived from multiple HLA allotypes. (A)** The use of LFSE-assisted MHCpLogics workflow to perform allelic-based cluster analysis, resulted in 13 (sub)clusters after the optimization of the number of clusters. **(B)** The utilization of the three approaches (i.e., *pchcat1*, *pchcat2*, and *pchcat3*) of the SMI function to show performance in macro clustering. |

| 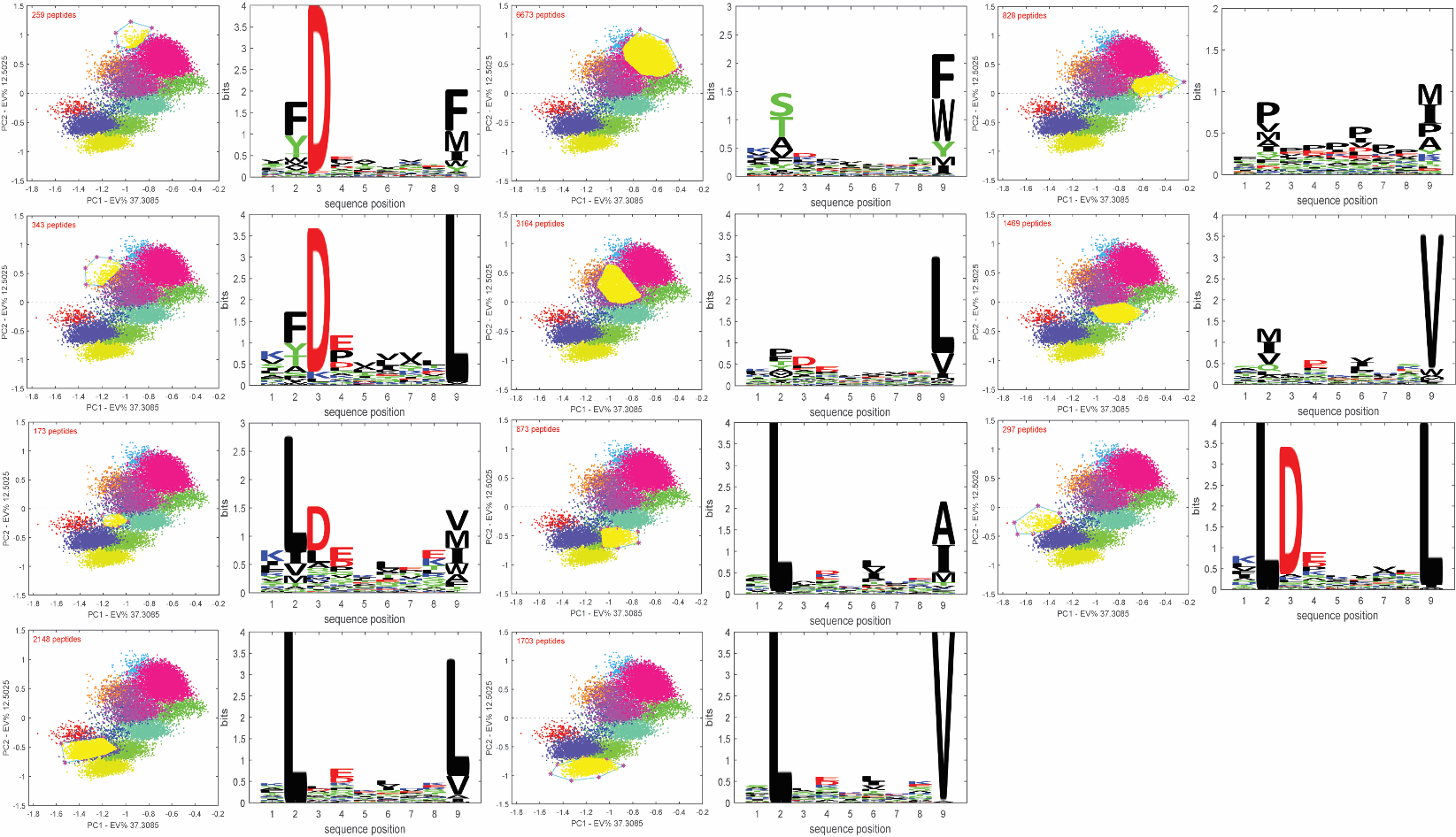 |
| --- |
| **Figure S5. An analytical approach to combine data visualization and cluster analysis outputs in MHCpLogics.** The LM-MEL-33 dataset was analyzed and the data visualized using a gating strategy across the data space. Combining the cluster analysis output and peptide score data allows visualization of specific clusters. |

| 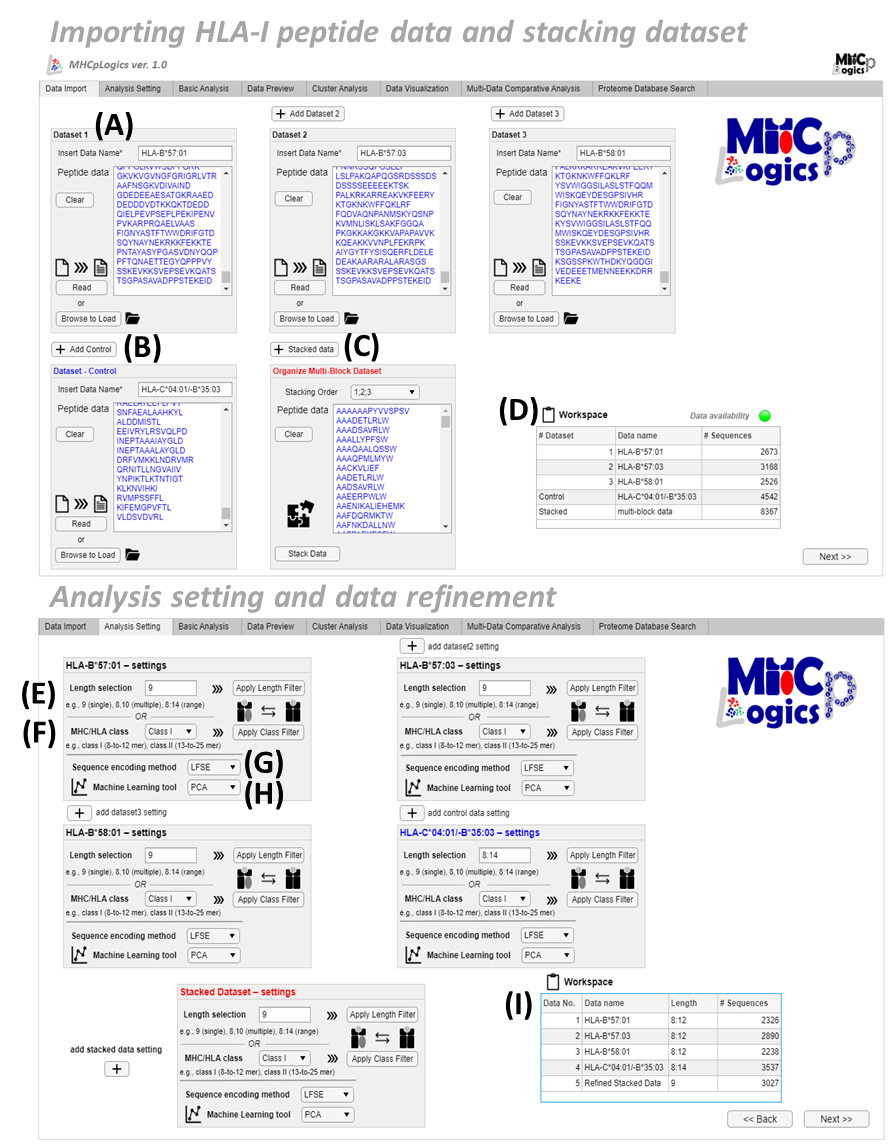 | | | |
| --- | --- | --- | --- |
| **Figure S6. GUI design for** **data import and analysis setting. (A)** Import up to three datasets. First, insert the data name, copy-paste the peptide data, and push “Read” to import the data. Users can also browse to select (over user’s computer) and load the data by choosing *txt or *csv files. **(B)** Users can also add a control dataset (optional and not mandatory). **(C)** Stack the datasets for comparative analysis. **(D)** Workspace of the imported data showing general information about the datasets (i.e., Data No., Data name, and #sequences). **(E)** Length-based sequence filtering. **(F)** MHC class-based sequence filtering. **(G)** Set the sequence scoring function (encoding method). **(H)** Set the machine learning tool, i.e., PCA, kPCA, MDS, t-SNE, and PCA-t-SNE. **(I)** Checking the refined datasets in the workspace. | | | |
|  | | |  |
| 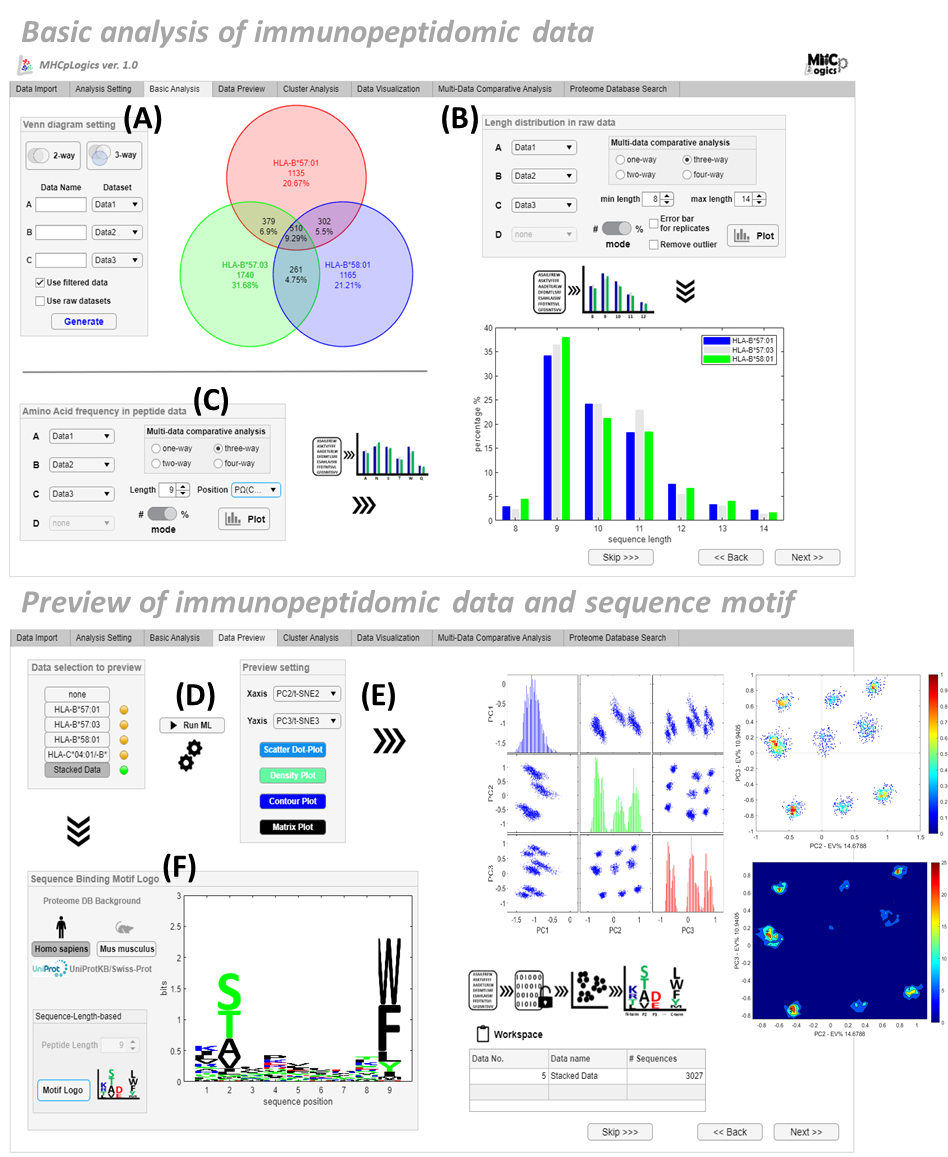 | | | |
| **Figure S7. GUI design for** **basic analysis and data preview. (A)** Set up the datasets and number of sets in Venn diagram (in the current version as 2-way and 3-way). **(B)** Set up and select datasets to analyze length distribution. This allows comparative analysis of the length distributions for multiple datasets. **(C)** The amino acid frequency analysis was set up at anchor residues in a multi-data comparative mode. **(D)** Selection of the data and running ML algorithm to preview data before major analyses (cluster analysis and data visualization). **(E)** Using different plotting-based visualization means (e.g., scatter, density, contour, and matrix plot) to preview data. **(F)** Checking sequence binding motif for each dataset for peptide data with different lengths. | | | |
| 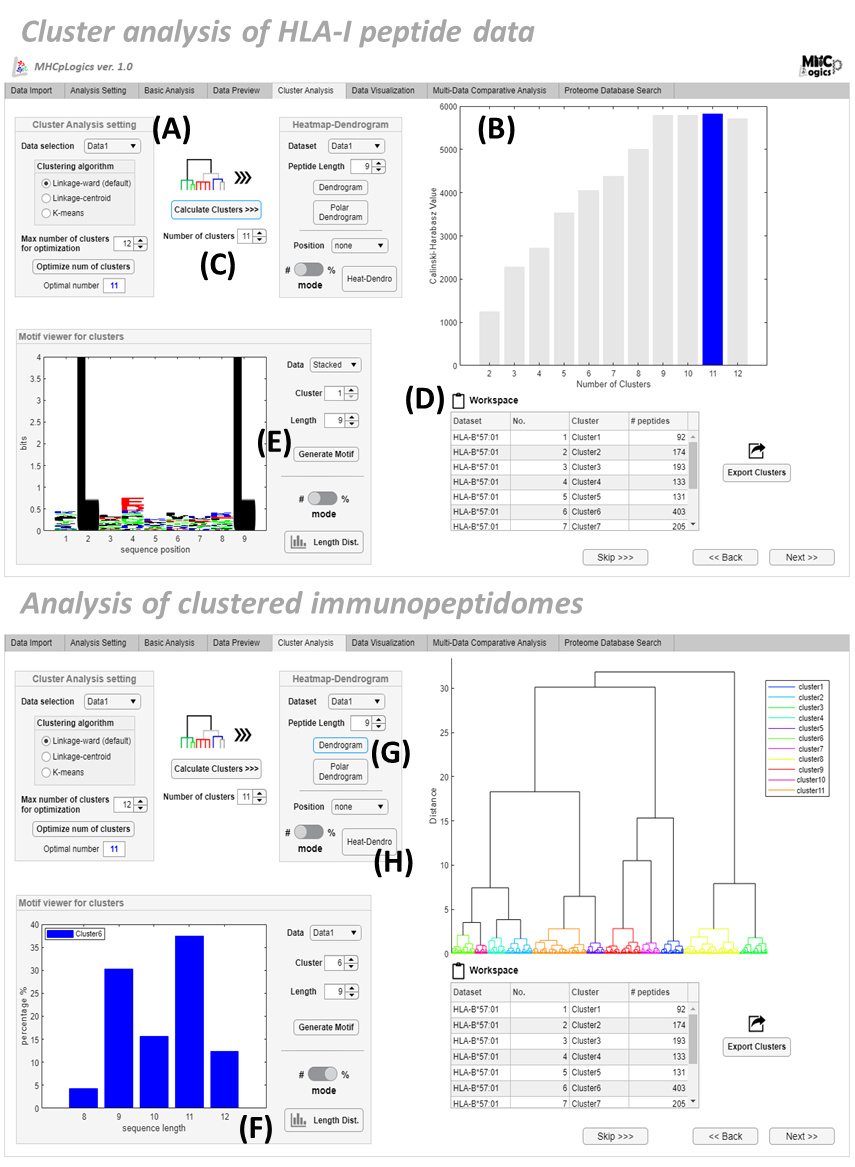 | |  |  |
| **Figure S8. GUI design for cluster analysis of immunopeptidomes. (A)** Set up the datasets, clustering algorithm, and the maximal number of clusters (based on our initial estimation) to optimize the number of clusters. **(B)** Checking the optimal number of clusters evaluated by Calinski-Harabasz Criterion scores. **(C)** Perform cluster analysis. **(D)** Checking the output of the clustering analysis by the information of the generated clusters on the workspace. **(E)** Checking sequence binding motif per cluster. **(F)** Analysis of the length distribution for each cluster (with multiple lengths). **(G)** Drawing regular and polar dendrograms to show similarities between the clusters. (H) Plotting heat-dendrogram to compare clusters based on amino acid frequencies at different residues. | |  |  |

| 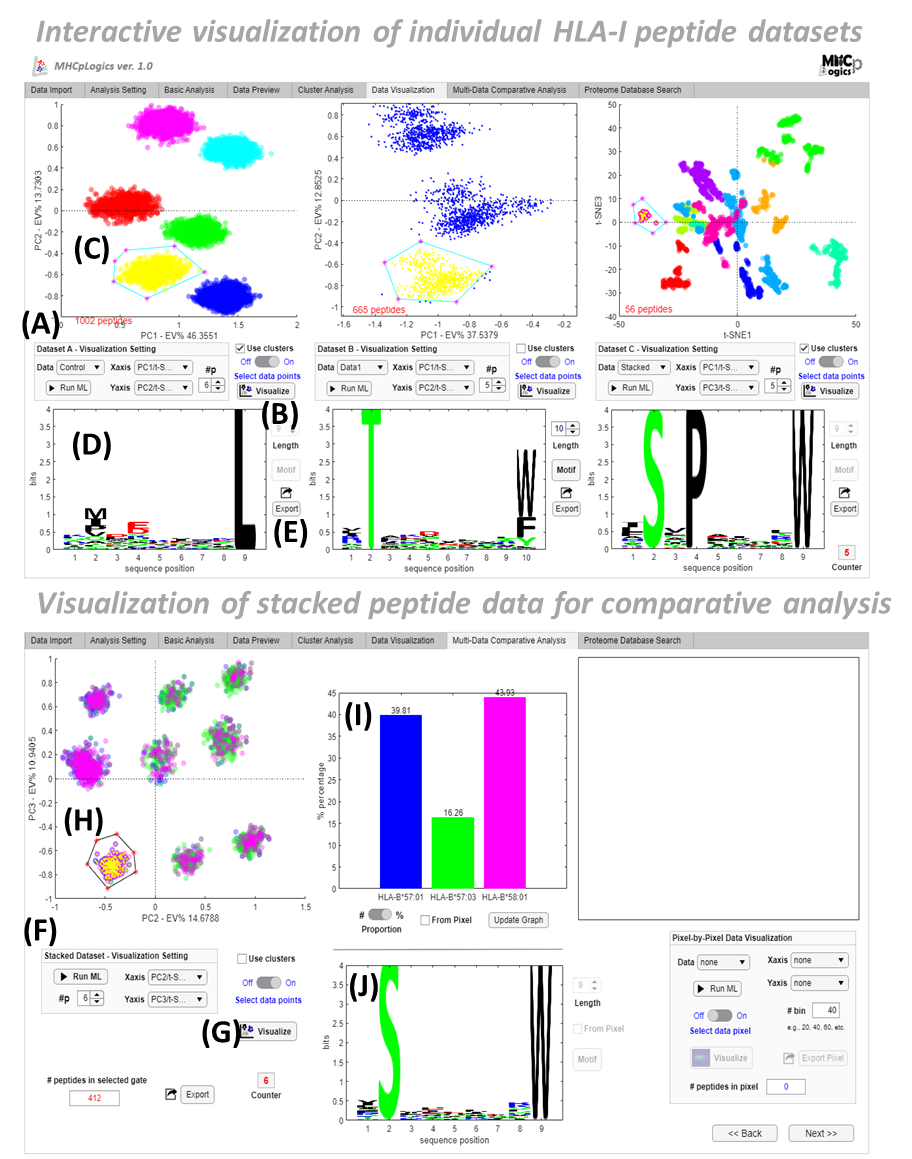 |  |
| --- | --- |
| **Figure S9. GUI design for data visualization and comparative analysis of immunopeptidomes. (A)** Set up the datasets, run ML algorithm and visualization parameters (detailed in the tutorial). **(B)** Visualize data assisted by gating strategy. **(C)** Selection of the gate points on the data space to explore immunopeptidomes with specialized features. **(D)** Checking sequence motif for the selected gate (sub-cluster). **(E)** Export the selected sub-cluster. **(F)** Running ML on the stacked data for multi-data comparative analysis and setting up the data visualization parameters. **(G)** Visualize the stacked data to analyze and compare individual datasets. **(H)** Selecting the gate points on the stacked data space. **(I)** Assessment of proportions of peptides originating from a specific dataset for the selected gate. **(J)** Checking sequence motif for the selected sub-cluster. |  |
| 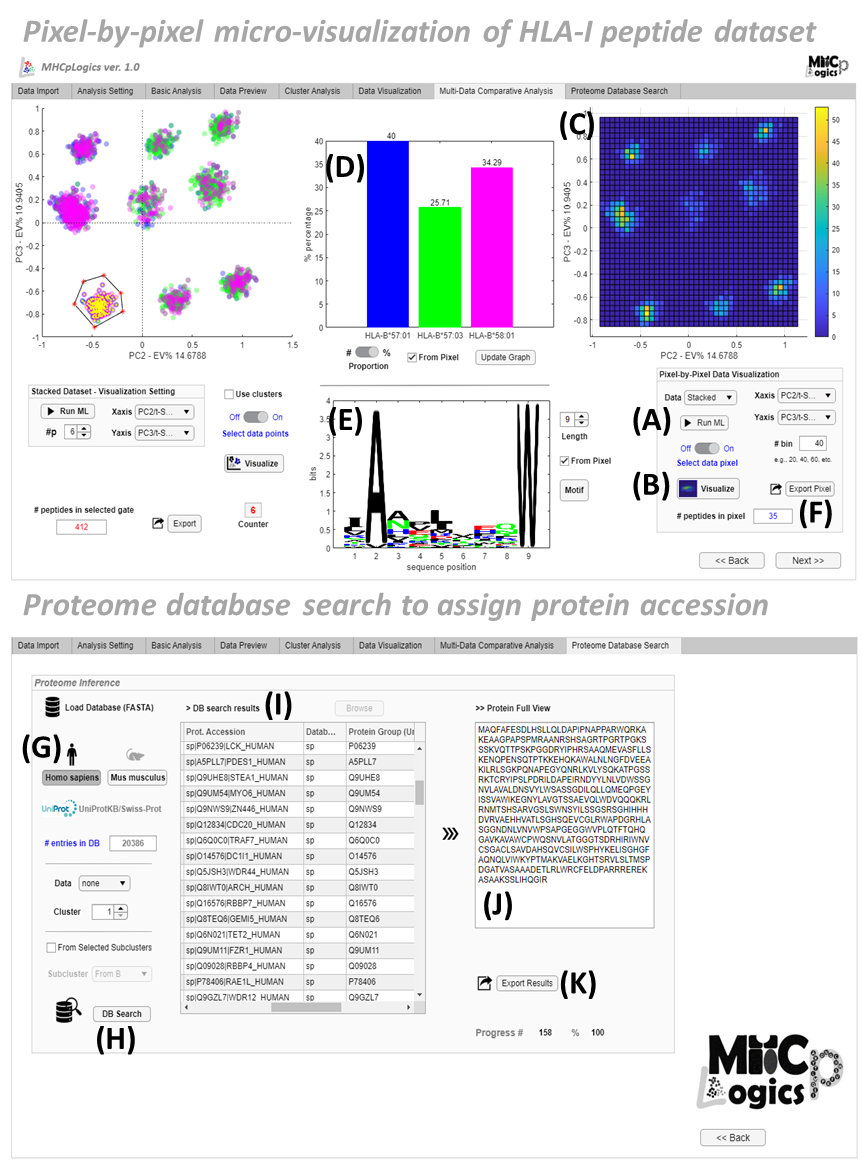 | |
| **Figure S10. GUI design for pixel-by-pixel micro visualization of immunopeptidomes and database search for protein inference. (A)** Set up the datasets, run ML algorithm and micro-visualization parameters (detailed in the tutorial). **(B)** Visualize pixelated data assisted using a gating strategy. **(C)** Selection of the pixel on the data space to explore specific sequence space within immunopeptidomes. **(D)** Assessment of proportions of peptides originated from a specific dataset for the selected pixel. **(E)** Checking sequence motif for the selected gate (sub-cluster). **(F)** Export the selected pixel data. **(G)** Setting up the proteome database and selecting the peptide cluster (or sub-cluster) for protein source inference. **(H)** Run the database search to find corresponding protein accessions for the selected (sub)clusters of the HLA peptides. **(I)** View the results and protein accessions per peptide as a list. **(J)** Checking the full protein sequence upon selecting in the “DB search results.” **(K)** Export the DB search results. | |
